# Interferon alpha and beta receptor 1 knockout in human embryonic kidney 293 cells enhances the production efficiency of proteins or adenoviral vectors related to type I interferons

**DOI:** 10.3389/fbioe.2023.1192291

**Published:** 2023-07-05

**Authors:** Aro Kim, Jong-Hyeon Park, Min Ja Lee, Su-Mi Kim

**Affiliations:** Center for Foot-and-Mouth Disease Vaccine Research, Animal and Plant Quarantine Agency, Gimcheon-City, Gyeongsangbuk-do, Republic of Korea

**Keywords:** HEK 293 cells, CRISPR-Cas9, adenovirus vector, protein, IFNAR

## Abstract

Human embryonic kidney (HEK) 293 cells are widely used in protein and viral vector production owing to their high transfection efficiency, rapid growth, and suspension growth capability. Given their antiviral, anticancer, and immune-enhancing effects, type I interferons (IFNs) have been used to prevent and treat human and animal diseases. However, the binding of type I IFNs to the IFN-α and-β receptor (IFNAR) stimulates the expression of IFN-stimulated genes (ISGs). This phenomenon induces an antiviral state and promotes apoptosis in cells, thereby impeding protein or viral vector production. In this study, we generated an IFNAR subtype 1 knockout (KO) HEK 293 suspension (IFNAR-KO) cell line by using clustered regularly interspaced short palindromic repeats (CRISPR)/CRISPR-associated protein-9 nuclease (Cas9) technology. Upon treatment with human IFN-α, the IFNAR-KO cells showed a constant expression of ISGs, including 2ʹ-5ʹ-oligoadenylate synthetase 1 (*OAS1*), myxovirus resistance 1 (*Mx1*), protein kinase RNA-activated (*PKR*), and IFN-induced transmembrane protein 1 (*IFITM1*), when compared with the wild-type HEK 293 (WT) cells, wherein the ISGs were significantly upregulated. As a result, the titer of recombinant adenovirus expressing porcine IFN-α was significantly higher in the IFNAR-KO cells than in the WT cells. Furthermore, the IFNAR-KO cells continuously produced higher amounts of IFN-α protein than the WT cells. Thus, the CRISPR-Cas9-mediated IFNAR1 KO cell line can improve the production efficiency of proteins or viral vectors related to IFNs. The novel cell line may be used for producing vaccines and elucidating the type I IFN signaling pathway in cells.

## 1 Introduction

Human embryonic kidney (HEK) 293 cells are HEK cells with DNA fragments of adenovirus type five integrated into HEK cells (Graham et al., 1977; Louis et al., 1997). These cells are widely used in protein and viral vector production owing to their suspension growth in serum-free media, high efficiency of protein or viral vector production, and amenability to various transfection methods (Abaandou et al., 2021). Specifically, they have been used in the production of vaccine candidates based on recombinant adenoviruses, virus-like particles (VLPs), and influenza viruses (Le Ru et al., 2010; Shen et al., 2012; Thompson et al., 2015; Puente-Massaguer et al., 2022). In addition, large-scale production of recombinant proteins by transient or stable expression has been reported in HEK 293 suspension cells, such as HEK 293S and HEK 293F cells (Tan et al., 2021). The genetic modification of HEK 293 cells depending on protein characteristics has been conducted to enhance the production efficiency or quality of recombinant proteins (Reeves et al., 2002; Abaandou and Shiloach, 2018; Arena et al., 2019; Inwood et al., 2020).

Type I interferons (IFNs), which exert antiviral, anticancer, and immune-enhancing effects, are important in preventing and treating human and animal diseases (Aricò and Belardelli, 2012; Medrano et al., 2017). However, type I IFNs stimulate the expression of IFN-stimulated genes (ISGs) through the Janus kinase (JAK)-signal transducer and activator of transcription (STAT) pathway, which induces an antiviral state and promotes apoptosis in cells, thereby impeding protein or viral production (Chawla-Sarkar et al., 2003; Schindler et al., 2007). Induction of ISGs decreases the efficiency of viral production in cells, and blocking the JAK-STAT pathway can increase viral efficiency (Gavegnano et al., 2017). In addition, cell apoptosis can adversely affect protein production; thus, slowing down this process can increase protein production efficiency (Arena et al., 2019). The process by which IFNs stimulate cells through the JAK-STAT pathway begins when an IFN binds to the IFN-α and-β receptor (IFNAR) in the cell membrane. Thus, the *IFNAR* gene can be a potential target for genetic manipulation to increase the production efficiency of viral vectors or proteins in cells.

RNA interference (RNAi), mainly used in the production of gene knockout (KO) cell lines, often results in partial or incomplete KO of the target gene (Malina et al., 2013). Clustered regularly interspaced short palindromic repeats/CRISPR-associated protein 9 (CRISPR/Cas9) technology overcomes these disadvantages and enables the continuous and complete KO of genes; thus, this system can be used to produce genetically engineered cell lines. Cas9 nuclease, a protein that causes genetic modification by cutting DNA, is used along with a single guide RNA (sgRNA) that binds to the target gene and guides the Cas9 protein (Cho et al., 2013). The Cas9 protein and sgRNA complex then specifically bind to the target sequence and recognize and truncate the protospacer adjacent motif sequence located 3ʹ of the target gene. During the repair process of the truncated part, insertion–deletion mutations (InDel) occur, and the expression of a specific gene is suppressed.

In the present study, we established a novel single cloned cell line by knocking out the *IFNAR1* gene in HEK 293S cells using the CRISPR/Cas9 technology. We applied the newly designed sgRNA and confirmed that it inhibited *IFNAR1* gene expression and ISG activation in the HEK 293S IFNAR1 KO cells (IFNAR-KO). In addition, the titer of recombinant adenovirus expressing IFN-α in the prepared cell line was compared with that in conventional HEK 293S wild-type (WT) cells. Furthermore, we compared and analyzed the production efficiency of the IFN-α protein in the novel HEK 293 cell line. Therefore, we tested the production efficiency of proteins or viral vectors and the mechanisms associated with type I IFN in IFNAR-KO cells.

## 2 Materials and methods

### 2.1 Reagents, cells, and viruses

HEK 293 suspension cells (Gibco 293F cells, Product no. 11625019) were purchased from Thermo Fisher Scientific (Waltham, MA, United States). The WT and IFNAR-KO cells were maintained in CD293 medium (Thermo Fisher Scientific) supplemented with L-glutamine (Thermo Fisher Scientific) at 37°C and 110 rpm in a shaking incubator with 5% CO_2_. Porcine kidney (LFBK) cells (Plum Island Animal Disease Center, Orient, NY, United States) were cultured in Dulbecco’s modified Eagle’s medium (Thermo Fisher Scientific) supplemented with 10% fetal bovine serum (pH 7.4; Atlas Biologicals, CO, United States) at 37°C with 5% CO_2_. FMDV O/SKR/2002 (GenBank accession No. **AH012984.2**) was isolated from the Republic of Korea by the Animal and Plant Quarantine Agency (APQA). Recombinant adenovirus expressing porcine IFN-α and IFN-γ (Ad-poIFNαγ) and recombinant baculovirus (BacMam) expressing porcine IFN-α (Bac-Con3N IFN-α) were obtained from previous studies (Kim et al., 2015; Kim et al., 2022).

### 2.2 Establishment of the IFNAR-KO cell line using the CRISPR/Cas9 system

A sgRNA was designed targeting the ACT​TTA​TCC​TGA​GGT​GGA​ACA​GG sequence of exon 2 region in IFNAR1 for the gene knockout (NCBI Gene ID:3454) and prepared as previously described (Kim et al., 2014). The *in vitro* transcribed sgRNA was supplied by ToolGen Inc. (Seoul, Republic of Korea) and incubated with recombinant Cas9 protein (ToolGen, Inc.) at room temperature for 10 min. HEK 293S cells were plated at 2 × 10^5^ cells/well in 24-well plates and then electroporated with the Cas9 protein pre-mixed with sgRNA using the Neon Transfection system (Thermo Fisher Scientific) to introduce double-strand breaks by using a ribonucleoprotein complex. The cells were plated in 96-well plates by using the limiting dilution method after 2 days of transfection, to obtain a single clone for each of the cells. After 3 weeks, the isolated cell colonies were analyzed using the T7E1 assay and targeted deep sequencing, as previously described (Kim et al., 2009). Briefly, the genomic DNAs of the isolated cell clones were separated using a genome isolation kit (GeneAll, Seoul, Republic of Korea) in accordance with the manufacturer’s instructions. PCR amplicons, including nuclease target sites, were generated using the following primers (forward, 5ʹ-TGT GCT GGG AGC AAT CAT TA-3ʹ; reverse, 5ʹ-GAA GCT GGA ACA CCC AGA AG-3ʹ). The PCR amplicons were denatured and annealed to form heteroduplex DNA using a thermocycler, digested with T7 endonuclease 1 (ToolGen Inc.) for 30 min at 37°C, and then analyzed by agarose gel electrophoresis. The positive clones selected in the T7E1 assay were analyzed for their InDel patterns through targeted deep sequencing as previously described (Kim et al., 2014). Equal amounts of PCR amplicons were subjected to paired-end read sequencing using MiSeq (Illumina Inc., San Diego, CA, United States).

### 2.3 Western blotting analysis

The protein expression levels of human IFNAR1 were compared between the WT and the IFNAR-KO cells by Western blotting, as previously described (Kim et al., 2014). The 1: 1,000 dilution of anti-IFNAR1 rabbit monoclonal antibody (Abcam, Cambridge, United Kingdom) or 1:2000 dilution of anti-beta actin rabbit monoclonal antibody (Abcam) was used as the primary antibody. The 1:2000 dilution of goat anti-rabbit IgG H&L (horseradish peroxidase [HRP]; Abcam) was used as the secondary antibody. Proteins were detected using Pierce ECL Western blotting substrate (Thermo Fisher Scientific) and visualized with an Azure C600 imaging system (Azure Biosystems, Dublin, CA, United States).

### 2.4 Measurement of mRNA level of ISGs

The WT and IFNAR-KO cells seeded in 24-well plates were inoculated with human IFN-α protein (PBL Assay Science, Piscataway, NJ, United States) with 10^5^ units per well. The cells were collected at 0, 6, 24, and 48 hpi and then subjected to RNA extraction, DNase I treatment, and SYBR Green real-time RT-PCR, following previously described methods (Kim et al., 2010). Quantitative real-time RT-PCR was performed to measure the mRNA levels of human *OAS*, *Mx1*, *PKR*, *IFITM1*, and human glyceraldehyde 3-phosphate dehydrogenase (*GAPDH*) as an internal control (Supplementary Table S1). Primer sequences were obtained from previous studies (Xing et al., 2015; Zhou et al., 2019) and synthesized by Macrogen Inc. (Seoul, Republic of Korea).

### 2.5 Measurement of cell viability and CPE percentage

A mixture of trypan blue (Merck, Burlington, MA, United States) and cells was loaded onto a cell counting slide (Bio-Rad Laboratories Inc., Hercules, CA, United States), and cell viability was measured using a TC20 Automated Cell Counter (Bio-Rad Laboratories Inc.). The percentage of CPE was calculated as 100—percentage of cell viability.

### 2.6 Measurement of the recombinant adenovirus production efficiency

The WT and the IFNAR-KO cells (2 × 10^6^/mL) were infected with Ad-porcine-IFNαγ at a Multiplicity of infection (MOI) of five and cultured at 37°C in a shaking incubator with 5% CO_2_. Supernatants were collected at 0, 24, 40, 48, and 72 h post-infection and virus titration was performed. Viral titers were calculated using the Spearman-Karber method with 50% tissue culture infective doses (TCID_50_) (Ramakrishnan, 2016). To measure the viral production efficiency in various passaged cells, the supernatants were collected at 72 h post-infection and virus copy numbers were determined using the Adeno-X qPCR titration kit (TaKaRa Bio Inc., Mountain View, CA, United States).

### 2.7 Measurement of protein production efficiency

The WT and IFNAR-KO cells (2 × 10^6^/mL) were infected with Bac-Con3N IFN-α at a multiplicity of infection (MOI) of 50 and cultured at 37°C in a shaking incubator with 5% CO_2_. Sodium butyrate was added to the suspension culture to a final concentration of 5 mM, and 2 mL of CD293 medium was added at 2- and 4-days post baculovirus inoculation to enhance the efficiency of protein expression using the baculovirus (BacMam) system in mammalian cells (Guo et al., 2010). Supernatants were collected daily for five consecutive days, and the quantity of porcine IFN-α protein was measured using porcine IFN-α ELISA (PBL Assay Science).

### 2.8 FMDV-based IFN biological assay

The FMDV-based IFN biological assay was performed, according to a previously described method (Kim et al., 2022). LFBK cells were grown to 90% confluence and exposed to 2-fold dilutions of porcine IFN-α. After 24 h, the supernatants were removed, and the cells were inoculated with 250TCID_50_ FMDV O/SKR/2002 for 1 h and incubated at 37°C for 48 h. The antiviral activity (units) was measured at the highest IFN-α dilution that induced 50% inhibition of CPE.

### 2.9 Statistical analysis

Unpaired *t*-tests were performed using the GraphPad Prism software (version 5.0; GraphPad Software, La Jolla, CA, United States). Statistical significance was set at *p* < 0.05.

## 3 Results

### 3.1 Establishment of a HEK 293S IFNAR1 KO cell line using the CRISPR/Cas9 system

**FIGURE 1:**
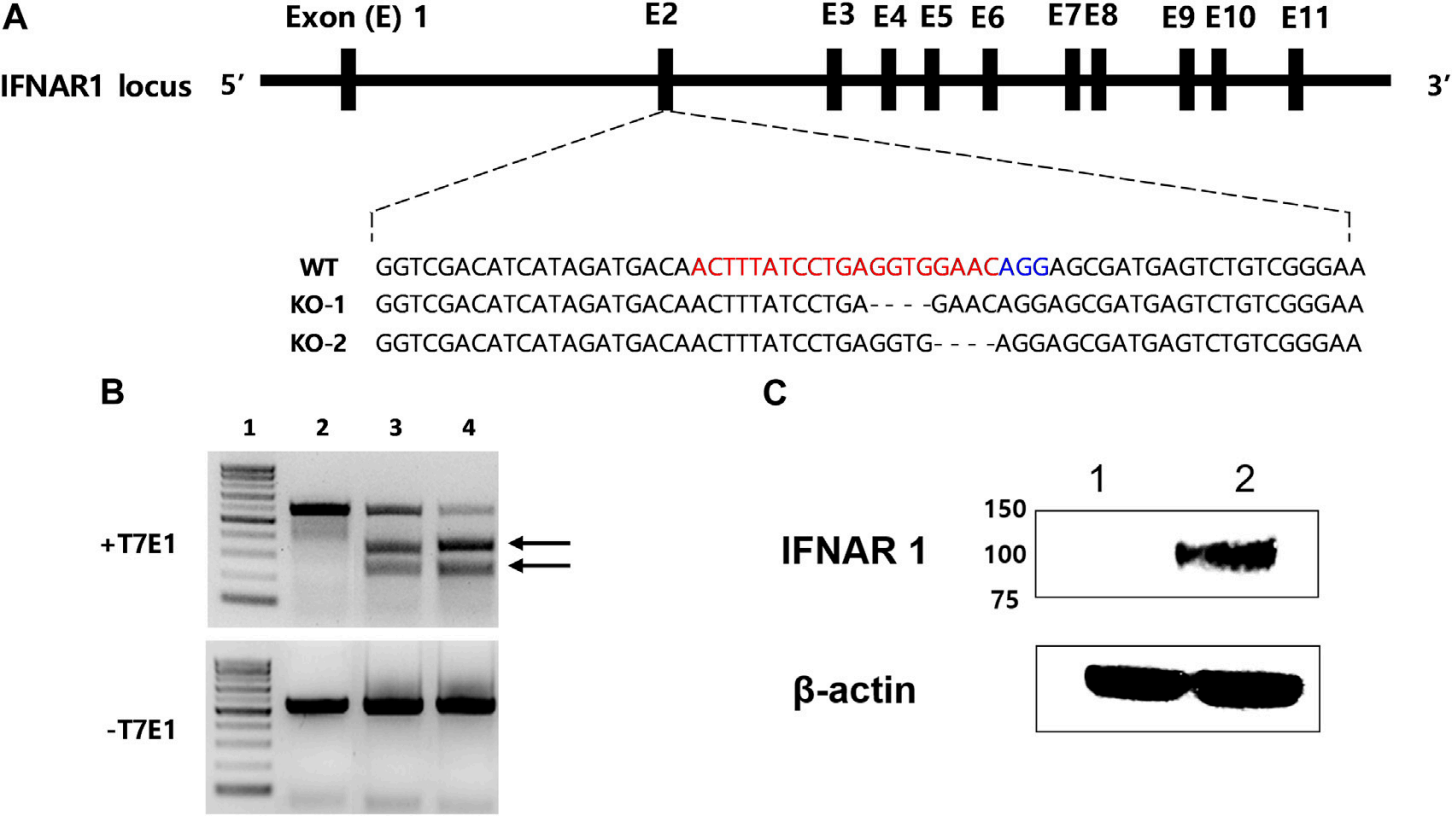
Generation of a 293S IFNAR1 KO cell line by using the CRISPR/Cas9 system. **(A)** The single guide (sg) RNA targeted sequences in the human IFNAR1 gene (red letters). The protospacer adjacent motif sequence AGG is highlighted in blue. The most frequent mutations were presented as KO-1 and KO-2. The dashed line represents deletion. **(B)** Electrophoresis results of the T7E1 assay. Arrows indicate bands cleaved by T7E1 enzyme. 1: size marker, 2: negative control, 3: sgRNA target sequence, 4: positive control. **(C)** Western blotting analysis of IFNAR1 expression in the HEK 293S and HEK 293S IFNAR1 KO cells. 1: HEK 293S IFNAR1 KO cells, 2: HEK 293S cells.

IFNAR-KO cells were generated using sgRNA targeting exon 2 of human IFNAR1 (Figure 1A). IFNAR1 deletions were confirmed through gene sequencing, as shown in KO-1 and KO-2 (Supplementary data sheet S2). In the T7 endonuclease 1 (T7E1) assay, when the corresponding sgRNA was used, a truncation pattern (expected band size of 229 and 321 bp) was confirmed, as in the positive control (Figure 1B). The IFNAR1 protein expression was compared between the WT and IFNAR-KO cells by Western blotting to confirm the silencing of IFNAR1 expression in the established cell line (Figure 1C). The band of glycosylated human IFNAR1 (estimated size of 90–130 kDa) was detected in the WT cells, but not in the IFNAR-KO cells. These results confirmed that *IFNAR1* gene expression was knocked out and the cell line was successfully established.

### 3.2 Inhibition of ISG activation in IFNAR-KO cells

**FIGURE 2:**
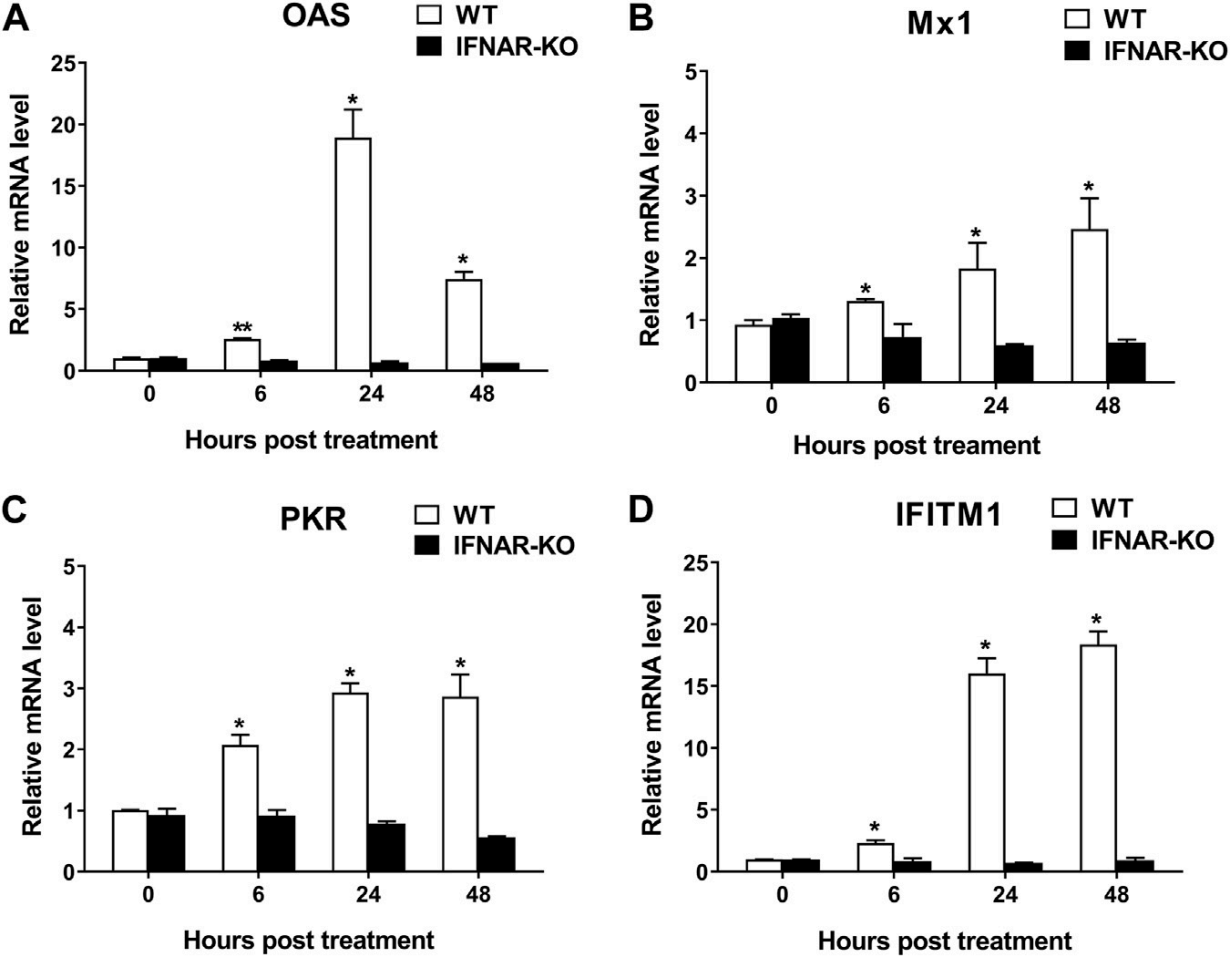
Blocking of interferon-stimulated gene (ISG) activation by the treatment of human interferon alpha in 293S IFNAR1 KO cells. Expression levels of OAS1 **(A)**, Mx1 **(B)**, PKR **(C)**, and IFITM1 **(D)** in HEK 293S (WT) and HEK 293S IFNAR1 KO (IFNAR-KO) cells treated with human interferon alpha protein of 10^5^ units/well were measured using quantitative real-time RT-PCR. Three independent experiments were performed. Error bars indicate standard deviations (SDs) from the mean. Unpaired *t*-test was performed to identify statistically significant differences. ^*^ *p* < 0.05, ^**^ *p* < 0.01.

To confirm the inhibition of IFN-α and -β receptors in the IFNAR-KO cells, we compared the mRNA levels of ISGs following treatment with human IFN-α protein in the IFNAR-KO and WT cells (Figure 2). The mRNA levels of ISGs, including 2ʹ-5ʹ-oligoadenylate synthetase (*OAS*), myxovirus resistance 1 (*Mx1*), protein kinase RNA-activated (*PKR*), and IFN-induced transmembrane protein 1 (*IFITM1*), significantly increased after treatment with human IFN-α in the WT cells at 6, 24, and 48 h post-treatment (*p* < 0.05). However, the mRNA levels of the corresponding genes did not increase in the IFNAR-KO cells.

### 3.3 Enhanced recombinant adenoviral titer in IFNAR-KO cells

**FIGURE 3:**
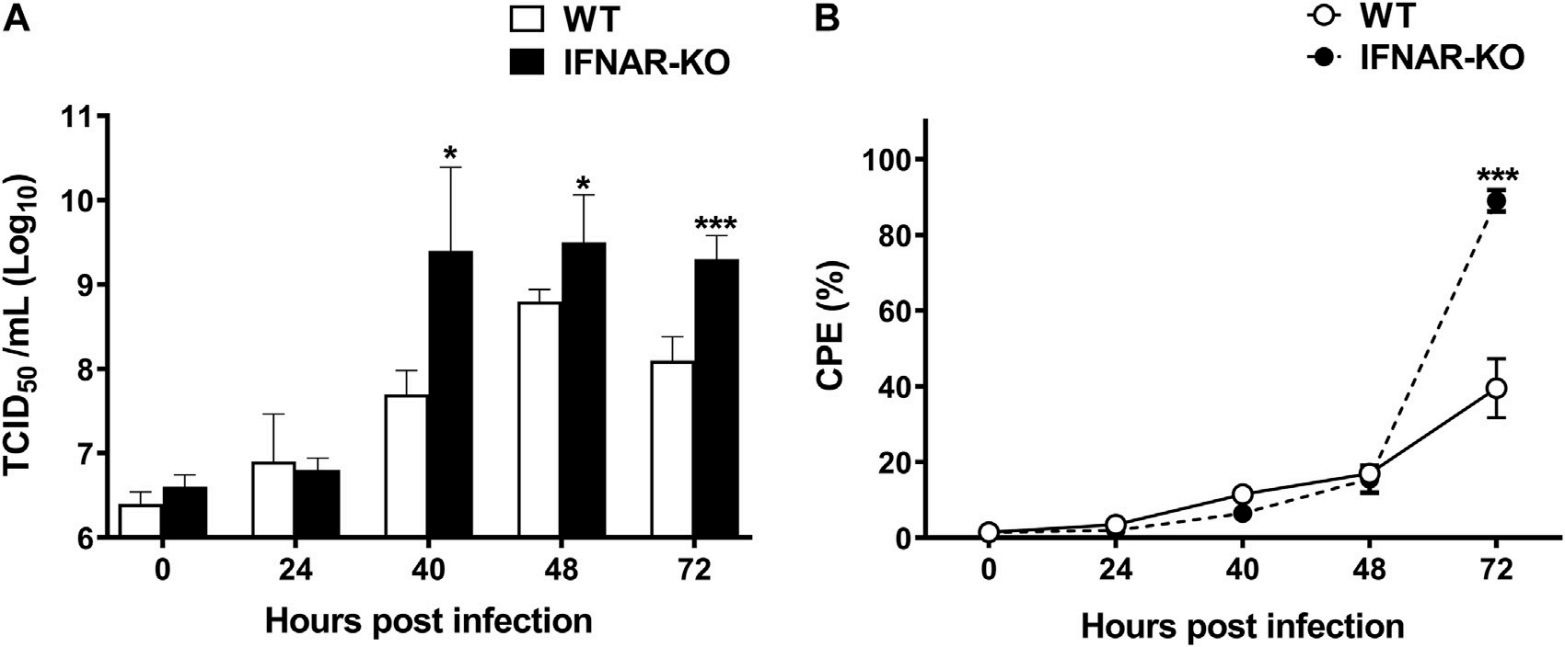
Enhanced production efficiency of Ad-IFNαγ virus in HEK 293S IFNAR1 KO cells. HEK 293S (WT) and HEK 293S IFNAR1 KO (IFNAR-KO) cells were infected with recombinant adenovirus expressing porcine IFNα and IFN-γ (Ad-IFNαγ virus) at a multiplicity of infection (MOI) of 5, and virus titration was performed using supernatants collected at 0, 24, 40, 48, and 72 h post infection (hpi) **(A)**. Cell viability was measured using a TC20 Automated Cell Counter. Percentage of cells with cytopathic effect (CPE) were calculated as 100—percentage of cell viability **(B)**. Three independent experiments were performed. Error bars indicate SDs from the mean. Unpaired *t*-test was performed to identify statistically significant differences. ^*^ *p* < 0.05, ^***^ *p* < 0.005.

To test the production efficiency of Ad-IFN-αγ in IFNAR-KO cells, we compared the virus titers in IFNAR-KO cells and WT cells. At 40–72 h post Ad-IFNαγ inoculation (hpi), the titers of recombinant adenovirus simultaneously expressing IFN-α and IFN-γ (Ad-IFNαγ) were significantly higher in the IFNAR-KO cells than in the WT cells (*p* < 0.05, Figure 3A). Ad-IFNαγ production was approximately 100-fold higher in the IFNAR-KO cells than in the WT cells at 40 hpi. In addition, a significant difference in cytopathic effect (CPE) was found between the IFNAR-KO and WT cells at 72 hpi (*p* < 0.005). More than 90% of the IFNAR-KO cells showed CPE, whereas only approximately 30% of the WT cells showed CPE at 72 hpi (Figure 3B).

### 3.4 Enhanced porcine IFN-α protein production in IFNAR-KO cells infected with BacMam expressing IFN-α (Bac-Con3N IFN-α)

**FIGURE 4:**
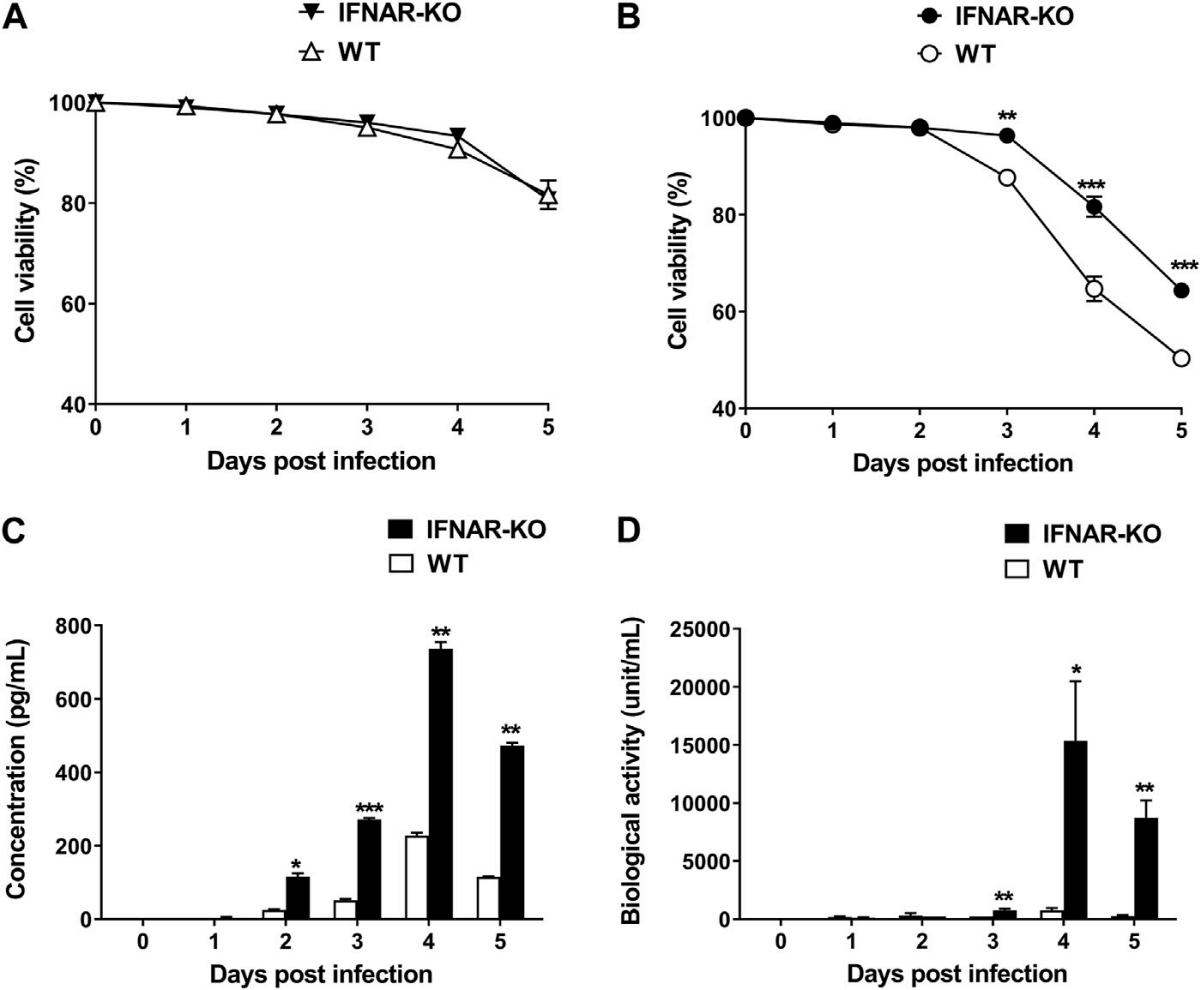
Increase in porcine IFN-α protein production efficiency in HEK 293S IFNAR1 KO cells. HEK 293S (WT) and HEK 293S IFNAR1 KO (IFNAR-KO) cells were infected with Bac-Con3N IFN-α at a multiplicity of infection (MOI) of 50, and supernatants were collected daily for five consecutive days. Viabilities of the WT and IFNAR-KO cells without Bac-Con3N IFN-α **(A)** and with Bac-Con3N IFN-α inoculation **(B)**. Amount of porcine IFN-α protein of supernatants measured using porcine IFN-α ELISA **(C)**. Biological activity of supernatants measured using FMDV-based IFN biological assay **(D)**. Three independent experiments were performed. Error bars indicate SDs from the mean. Unpaired *t*-test was performed to identify statistically significant differences. ^*^ *p* < 0.05, ^**^ *p* < 0.01, ^***^ *p* < 0.005.

To test the production efficiency of IFN-α protein in IFNAR KO cells, we compared the expression level of this protein in IFNAR-KO cells and WT cells. The viabilities of the WT and the IFNAR-KO cells were consistent for up to 5 days of culture (Figure 4A). At 3 days post inoculation with Bac-Con3N IFN-α, the IFNAR-KO cells showed a significantly higher viability than the WT cells (*p* < 0.01, Figure 4B). Moreover, IFN-α protein production was consistently higher in the IFNAR-KO cells than in the WT cells at 2–5 days post baculovirus inoculation (*p* < 0.05). At 4 days after inoculation, when the protein production was at its highest, the protein production in the WT cells was less than 40% than that in the IFNAR-KO cells (Figure 4C). In an FMDV-based IFN biological assay, IFN production was significantly higher in the IFNAR-KO cells than in the WT cells at 3–5 days post baculovirus inoculation, and the highest units/mL of IFN was obtained in the IFNAR-KO cells at 4 days post infection (*p* < 0.05, Figure 4D).

### 3.5 IFNAR-KO cells stably produced enhanced adenoviral titer over passages

**FIGURE 5:**
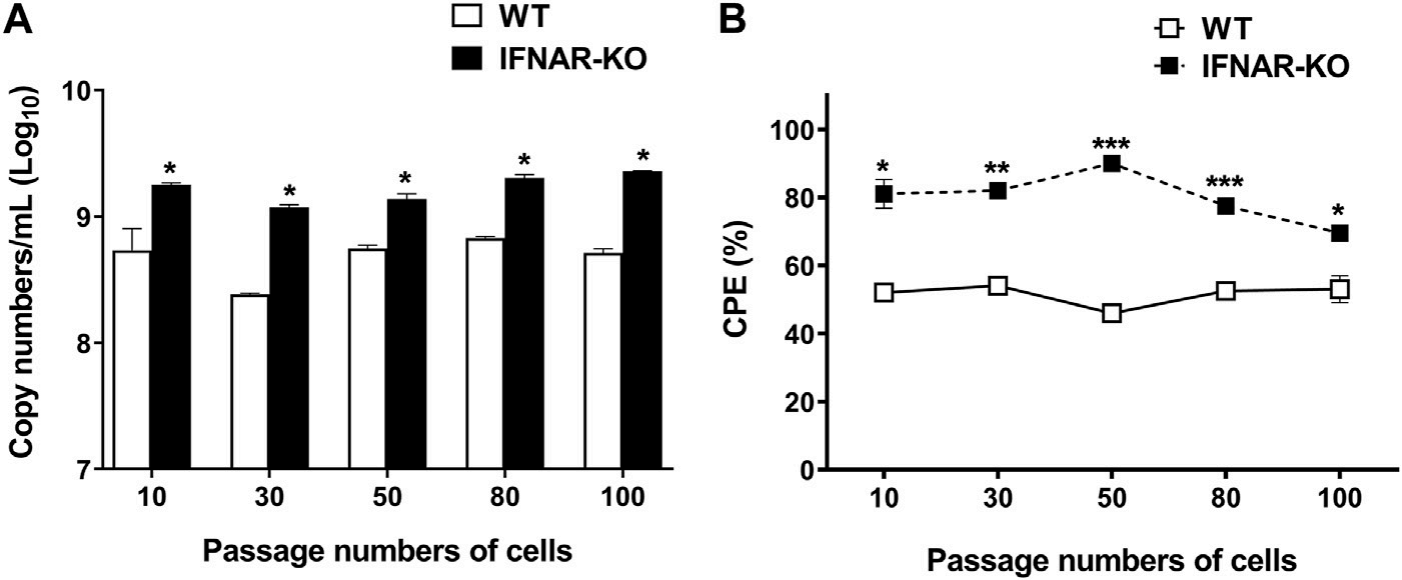
Effect of passage number of cells on adenovirus production efficiency. HEK 293S (WT) and HEK 293S IFNAR1 KO (IFNAR-KO) cells passaged 10, 30, 50, 80, and 100 times were infected with Ad-porcine-αγ at a multiplicity of infection (MOI) of 5, and supernatants were collected at 72 h post-infection. DNA extraction and quantitative real-time PCR were performed using the supernatants to assay for adenovirus DNA replication **(A)**. Percentage of cells with cytopathic effect (CPE) were calculated as 100 − percentage of cell viability. Cell viability was measured using a TC20 Automated Cell Counter **(B)**. Unpaired *t*-test was performed to identify statistically significant differences. ^*^ *p* < 0.05, ^**^ *p* < 0.01, ^***^ *p* < 0.005.

On evaluating adenovirus productivity according to the cell passages, it was found that the IFNAR-KO cells had a higher virus productivity than the WT cells for up to 100 passages (*p* < 0.05, Figure 5A). The viral copy numbers produced in the IFNAR-KO cells from passages 10 to 100 were not significantly different. In addition, the ratio of cells showing CPE at 72 hpi was also consistently higher in the IFNAR-KO cells than in the WT cells for up to 100 passages (*p* < 0.05, Figure 5B).

## 4 Discussion

In this study, we established a novel HEK 293 cell line with enhanced production efficiency of viral vectors or proteins by using the CRISPR-Cas9 technology. Modification of HEK 293 cells is valuable as these cells are widely utilized for the production of viral vectors, such as adenoviral and adeno-associated viral vectors (Tan et al., 2021). The HEK 293 cell line was established by transforming human embryonic kidney cells with sheared adenovirus type five E1A and E1B genes (Graham et al., 1977). This cell line has several advantages, such as high transfection efficiency, rapid growth rate, and suspension culture growth for manufacturing vaccines or proteins (Tan et al., 2021). Therefore, several FDA-approved recombinant protein or viral vector therapeutics, such as NUWIQ^®^ (coagulation factor VII), Luxturna^®^ (AAV-based RPE65 gene therapy), and Kymriah^®^ (CD-19-directed CAR T therapy), have been produced in HEK 293 cells.

Genetic modifications in established cell lines are widely studied because modifying cell characteristics can improve the production efficiency of viruses. Previous studies found that *IFNAR* gene knockdown in avian cell lines and modification in Vero cells using CRISPR-Cas9 directly enhanced the replication of influenza virus or poliovirus, respectively, for vaccine production (Carvajal-Yepes et al., 2015; van der Sanden et al., 2016). In the present study, we used a novel sgRNA sequence to target exon 2 of the IFNAR1 region and observed IFNAR1 gene KO (Figure 1). CRISPR-Cas9 technology is significantly more precise and stable than small interfering RNA and easier to handle when compared to other gene-editing technologies, such as zinc-finger nucleases and transcription activator-like effector nucleases (Shin et al., 2016; Li et al., 2020). In the present study, we used CRISPR-Cas9 technology for effective and prolonged gene editing of the cell line. In this study, we obtained 210 single cell clones and 107 cell clones that were positive according to the T7E1 assay (data not shown). The positive colonies showed truncated patterns similar to the positive control (Figure 1B). The positive control of T7E1 assay was obtained from the HEK 293 cells transfected with Cas9 protein and the other sgRNA (ACA​GGA​GCG​ATG​AGT​CTG​TC) guaranteed for IFNAR-KO in the HAP1 (human near-haploid) cell line. Among the positive colonies, 29 single cell clones were analyzed using targeted deep sequencing. Only one cell clone had an InDel frequency of 100% and it was used in this study (Supplementary data sheet S2). We observed that the enhanced production of adenovirus was maintained up to 100 passages of the IFNAR-KO cells (Figure 5) although the InDel pattern or protein expression of the high passaged cells should be analyzed in further studies.

IFNAR1 and IFNAR2 membrane proteins form a heterodimer, and each IFNAR plays distinct roles in initiating JAK-STAT pathway-mediated type I IFN production (de Weerd et al., 2007; Shemesh et al., 2021). IFNAR1 is associated with tyrosine kinase 2 (TYK2), IFNAR2 is associated with JAK1 and TYK2, and JAK1 forms a functional signaling unit in the JAK/STAT pathway. Type I IFN signaling pathways are equally inactivated in IFNAR1-or IFNAR2-KO HeLa cells, whereas they remained functional in STAT-KO HeLa cells (Urin et al., 2019). In the present study, KO of exon 2 of IFNAR1 was effective in blocking ISG stimulation by type I IFN in HEK 293 cells (Figure 2). Furthermore, IFNAR1 KO did not exert negative effects on the growth rate or morphology of HEK 293 cells (data not shown). Therefore, we strongly believe that IFNAR1 could be a good target for the novel sgRNA used in this study to effectively eliminate IFN-induced activity.

The adenovirus expression system can induce type I IFN. However, the level is not sufficient to inhibit viral replication in HEK 293 cells or protein expression in target cells (Huarte et al., 2006; Nidetz et al., 2018). Therefore, recombinant adenovirus is suitable to be produced in HEK 293 cells and used as a delivery vector. However, type I IFNs expressed or induced by target genes of adenoviral vectors, such as Ad-IFNαγ or the adenovirus expressing VLPs, could interfere with the replication of recombinant adenovirus in HEK 293 cells because type I IFNs suppress adenoviral replication in cells (Zheng et al., 2016; Chikhalya et al., 2021). Zheng et al. (2016) reported that type I and type II IFNs downregulate the replication of human adenovirus (maximum 100-fold) through an evolutionarily conserved E2F binding site in the immediate early E1A gene transcriptional region. We also observed that the titer of the recombinant adenovirus expressing human or porcine IFN-α/γ was significantly lower (≥50-fold) than that of the recombinant adenovirus negative control, and the adenoviral titer was recovered by treating the cells with the anti-IFN-α antibody to remove the IFN-α protein (data not shown). We previously demonstrated that Ad-porcine IFNαγ has significant antiviral effects against FMDV in cells and pigs (Kim et al., 2014; Kim et al., 2015). However, enhancing the productivity of adenovirus in the suspension culture was difficult because of the type I IFN-mediated inhibition of adenoviral replication. Therefore, we engineered an IFNAR1 knockout cell line to minimize the antiviral effect of IFNs. Suppression of *IFNAR* gene expression could effectively enhance viral replication because the stimulation of the JAK-STAT pathway by type I IFNs that induces ISG expression is inhibited (Palacio et al., 2020; Xiang et al., 2022). In the present study, type I IFNs did not stimulate ISG expression in the IFNAR-KO cells (Figure 2), thereby improving the productivity of Ad-IFNαγ (Figure 3). We propose that the IFNAR-KO cell line can be effectively applied in the production of vaccines using adenovirus-expressing VLPs or viral proteins or stimulating immune function by triggering IFN response (Appledorn et al., 2010; Ayithan et al., 2015; Beiss et al., 2022; Bhat et al., 2022).

HEK 293 cells are useful in the production of recombinant proteins. Various modifications have been conducted in these cells to increase their protein yields, nutrient utilization efficiency, and homogenous glycosylation (Abaandou et al., 2021). In addition, numerous studies employed gene engineering to delay the apoptosis of HEK 293 cells and increase the production efficiency of recombinant proteins (Zhang et al., 2017; Abbasi-Malati et al., 2019; Arena et al., 2019). Apoptosis-resistant cells may have increased culture longevity, viability, and recombinant protein expression. The downregulation of pro-apoptotic genes, such as Bcl-2 like protein 4 (*BAX*), Bcl-2 homologous antagonist/killer *(BAK*), *Caspase 3*, and allograft inflammatory factor 1 (*A1F1*), or the upregulation of anti-apoptotic genes, such as nuclear factor erythroid 2-related factor 2 (*NRF2*), have been reported. In the present study, we suggest an alternative approach for delaying cell apoptosis. We used the BacMam system expressing porcine IFN-α (Bac-Con3N IFN-α) to test the efficiency of IFN production in the IFNAR-KO cells when compared with the WT cells (Figure 4). In our previous study, we reported that Bac-Con3N IFN-α exerts significant antiviral and adjuvant effects against FMDV in pigs (Kim et al., 2022). BacMam is a baculovirus expression system driven by the cytomegalovirus immediate-early promoter in mammalian cells and is an effective transduction tool for protein expression in HEK 293 cells (Dukkipati et al., 2008; Goehring et al., 2014; Sung et al., 2014). In the present study, the IFN protein yield and viability of the IFNAR-KO cells significantly improved (Figure 3), suggesting that the apoptosis induced by type I IFNs was possibly minimized in these cells. IFNAR is a critical factor in apoptosis, as type I IFN binding to IFNAR activates the JAK-STAT pathway, leading to the expression of pro-apoptotic caspases. ISGs induced by type I IFNs also mediate apoptosis, which suppresses protein production (Thyrell et al., 2002; Chawla-Sarkar et al., 2003; Apelbaum et al., 2013). Hence, the established HEK 293S IFNAR1 KO cell line could be used for producing proteins that cause IFN-induced apoptosis.

In conclusion, we established a HEK 293 suspension cell line that is not affected by type I IFNs through *IFNAR1* gene knockout. The cell line can be effectively used to manufacture adenoviral vectors and IFN proteins. It can also be employed for the large-scale production of vaccines using live viruses, viral proteins, VLPs, viral vectors, and proteins related to IFNs or apoptosis. Furthermore, this cell line could be used to elucidate the type I IFN signaling pathway in cells.

## Data Availability

The raw sequencing data of IFNAR-KO cells are deposited in the NCBI Sequencing Read Archive (SRA accession no. ARR24966139).
