# Supplementary material for: Interferon alpha and beta receptor 1 knockout in human embryonic kidney 293 cells enhances the production efficiency of proteins or adenoviral vectors related to type I interferons
Source: Front Bioeng Biotechnol. 2023 Jul 5;11:1192291. doi: 10.3389/fbioe.2023.1192291 (PMC10355049; doi:10.3389/fbioe.2023.1192291)
Supplement: Supplementary file 1 [file Table1.DOCX]

**Supplementary Table 1.** Primers used in this study.

| **Gene** | **Primer/Probe** | **Sequence 5′ to 3′** | **Reference** |
| --- | --- | --- | --- |
| *GAPDH* | Forward primer | GTCTCCTCTGACTTCAACAGCG | Zhou et al. (2019) |
|  | Reverse primer | ACCACCCTGTTGCTGTAGCCAA |  |
| *Mx1* | Forward primer | GGCTGTTTACCAGACTCCGACA |  |
|  | Reverse primer | CACAAAGCCTGGCAGCTCTCTA |  |
| *IFITM1* | Forward primer | GGCTTCATAGCATTCGCCTACTC |  |
|  | Reverse primer | AGATGTTCAGGCACTTGGCGGT |  |
| *OAS* | Forward primer | AGGAAAGGTGCTTCCGAGGTAG | Xing et al. (2015) |
|  | Reverse primer | GGACTGAGGAAGACAACCAGGT |  |
| *PKR* | Forward primer | GAAGTGGACCTCTACGCTTTGG |  |
|  | Reverse primer | TGATGCCATCCCGTAGGTCTGT |  |
