# Supplementary material for: Interferon alpha and beta receptor 1 knockout in human embryonic kidney 293 cells enhances the production efficiency of proteins or adenoviral vectors related to type I interferons
Source: Front Bioeng Biotechnol. 2023 Jul 5;11:1192291. doi: 10.3389/fbioe.2023.1192291 (PMC10355049; doi:10.3389/fbioe.2023.1192291)
Supplement: Supplementary file 2 [file DataSheet1.PDF]

| No. | WT Sequence | Length | Type |
|---|---|---|---|
| | TGGAAAAAATCTAAAATCTCCTCAAAAAGTAGAGGTCGACATCATAGATGACAACTTTATCCTGAGGTGGAACAGGAGCGATGAGTCTGTCGGGAATGTGACTTTTTCATTCGATTATCAAAAGTATGTGACTCTACTTA | | |
| No. | **RGEN Treated Sequence** | **Length** | **Type** |
| 1 | TGGAAAAAATCTAAAATCTCCTCAAAAAGTAGAGGTCGACATCATAGATGACAACTTTATCCTGA----GAACAGGAGCGATGAGTCTGTCGGGAATGTGACTTTTTCATTCGATTATCAAAAGTATGTGACTCTACTTA | 136 | deletion |
| 2 | TGGAAAAAATCTAAAATCTCCTCAAAAAGTAGAGGTCGACATCATAGATGACAACTTTATCCTGAGGTG----AGGAGCGATGAGTCTGTCGGGAATGTGACTTTTTCATTCGATTATCAAAAGTATGTGACTCTACTTA | 136 | deletion |
| 3 | TGGAAAAAATCTAAAATCTCCTCAAAAAGTAGAGGTCGACATCATAGATGACAACTTTATCCTGA----GAACAGGAGCGATGAGTCTGTCGGGAATGTGACTTTTTCATTCGATTATCAAAAGTATGTGACTCTACTGA | 136 | deletion |
| 4 | TGGAAAAAATCTAAAATCTCCTCAAAAAGTAGAGGTCGACATCATAGATGACAACTTTATCCTGAGGTG----AGGAGCGATGAGTCTGTCGGGAATGTGACTTTTTCATTCGATTATCAAAAGTATGTGACTCTACTGA | 136 | deletion |
| 5 | TGGAAAAAATCTAAAATCTCCTCAAAAAGTAGAGGTCGACATCATAGATGACAACTTTATCCTGA----GAACAGGAGCGATGAGTCTGTCGGGAATGTGACTTTTTCATTCGATTATAAAAAGTATGTGACTCTACTTA | 136 | deletion |
| 6 | TGGAAAAAATCTAAAATCTCCTCAAAAAGTAGAGGTCGACATCATAGATGACAACTTTATCCTGAGGTG----AGGAGCGATGAGTCTGTCGGGAATGTGACTTTTTCATTCGATTATAAAAAGTATGTGACTCTACTTA | 136 | deletion |
| 7 | CGGAAAAAATCTAAAATCTCCTCAAAAAGTAGAGGTCGACATCATAGATGACAACTTTATCCTGA----GAACAGGAGCGATGAGTCTGTCGGGAATGTGACTTTTTCATTCGATTATCAAAAGTATGTGACTCTACTTA | 136 | deletion |
| 8 | TGGAAAAAATCTAAAATCTCCTCAAAAAGTAGAGGTCGACATCATAGATGACAACTTTATCCTGA----GAACAGGAGCGATGAGCCTGTCGGGAATGTGACTTTTTCATTCGATTATCAAAAGTATGTGACTCTACTTA | 136 | deletion |
| 9 | TGGAAAAAATCTAAAATCTCCTCAAAAAGTAGAGGTCGACATCATAGATGACAACTTTATCCTGAGGTG----AGGAGCGATGAGTCTGTCGGGAATGTGACTTTTTCATTCGATTATCAAAAGTATGTGACTCTGCTTA | 136 | deletion |
| 10 | TGGAAAAAATCTAAAATCTCCTCAAAAAGTAGAGGTCGACATCATAGATGACAACTTTATCCTGAGGTG----AGGAGCGATGAGTCTGTCGGGAATGTGACTTTTTCATTCGATTATCAAAAGTATGTGACTCTCCTTA | 136 | deletion |
| 11 | TGGAAAAAATCTAAAATCTCCTCAAAAAGTAGAGGTCGACATCATAGATGACAACTTTATCCTGA----GAACAGGAGCGATGAGTCTGTCGGGAATGTGACTTTTTCATTCGATTATCAAAAGTATGTGACTCTCCTTA | 136 | deletion |
| 12 | TGGAAAAAATCTAAAATCTCCTCAAAAAGTAGAGGTCGACATCATAGATGACAACTTTATCCTGAGGTG----AAGAGCGATGAGTCTGTCGGGAATGTGACTTTTTCATTCGATTATCAAAAGTATGTGACTCTACTTA | 136 | deletion |
| 13 | TGGAAAAAATCTAAAATCTCCTCAAAAAGTAGAGGTCGACATCATAGATGACAACTTTATCCTGAGGTG----AGGAGCGATGAGCCTGTCGGGAATGTGACTTTTTCATTCGATTATCAAAAGTATGTGACTCTACTTA | 136 | deletion |
| 14 | TGGAAAAAATCTAAAATCTCCTCAAAAAGTAGAGGTCGACATCATAGATGACAACTTTATCCTGAGGTG----AGGAGCGATGAGTCTGTCGGGAATGTGGCTTTTTCATTCGATTATCAAAAGTATGTGACTCTACTTA | 136 | deletion |
| 15 | TGGAAAAAATCTAAAATCTCCTCAAAAAGTAGAGGTCGACATCATAGATGACAACTTTATCCTGA----GAACAGGAGCGATGAGTCTGTCGGGAATGTGGCTTTTTCATTCGATTATCAAAAGTATGTGACTCTACTTA | 136 | deletion |
| 16 | TGGAAAAAATCTAAAATCTCCTCAAAAAGTAGAGGTCGACATCATAGATGACAACTTTATCCTGAGGTGG----GGAGCGATGAGTCTGTCGGGAATGTGACTTTTTCATTCGATTATCAAAAGTATGTGACTCTACTTA | 136 | deletion |
| 17 | TGGAAAAAATCTAAAATCTCCTCAAAAAGTAGAGGTCGACATTATAGATGACAACTTTATCCTGA----GAACAGGAGCGATGAGTCTGTCGGGAATGTGACTTTTTCATTCGATTATCAAAAGTATGTGACTCTACTTA | 136 | deletion |
| 18 | TGGAAAAAATCTAAAATCTCCTCAAAAAGTAGAGGTCGACATCATAGATGACAACTTTATCCTGAGGTG----AGGAGCGATGAGTCTGTTGGGAATGTGACTTTTTCATTCGATTATCAAAAGTATGTGACTCTACTTA | 136 | deletion |
| 19 | TGGAAAAAATCTAAAATCTCCTCAAAAAGTAGAGGTTGACATCATAGATGACAACTTTATCCTGAGGTG----AGGAGCGATGAGTCTGTCGGGAATGTGACTTTTTCATTCGATTATCAAAAGTATGTGACTCTACTTA | 136 | deletion |
| 20 | TGGAAAAAATCTAAAATCTTCTCAAAAAGTAGAGGTCGACATCATAGATGACAACTTTATCCTGA----GAACAGGAGCGATGAGTCTGTCGGGAATGTGACTTTTTCATTCGATTATCAAAAGTATGTGACTCTACTTA | 136 | deletion |
| 21 | TGGAAAAAATCTAAAATCTCCTCAAAAAGTAGAGGTCGACATCATAGATGACAACTTTATCCTGA----GAACAGGAGCGATGAGTCTGTCGGGAATGTGACTTTTTCATTTGATTATCAAAAGTATGTGACTCTACTTA | 136 | deletion |
| 22 | TGGAAAAAATCTAAAATCTCCTCAAAAAGTAGAGGTTGACATCATAGATGACAACTTTATCCTGA----GAACAGGAGCGATGAGTCTGTCGGGAATGTGACTTTTTCATTCGATTATCAAAAGTATGTGACTCTACTTA | 136 | deletion |
| 23 | TGGAAAAAATCTAAAATCTCTTCAAAAAGTAGAGGTCGACATCATAGATGACAACTTTATCCTGA----GAACAGGAGCGATGAGTCTGTCGGGAATGTGACTTTTTCATTCGATTATCAAAAGTATGTGACTCTACTTA | 136 | deletion |
| 24 | TGGAAAAAATCTAAAATCTCCTCAAAAAGTAGAGGTCGACATCATAGATGGCAACTTTATCCTGA----GAACAGGAGCGATGAGTCTGTCGGGAATGTGACTTTTTCATTCGATTATCAAAAGTATGTGACTCTACTTA | 136 | deletion |
| 25 | TGGAAAAAATCTAAAATCTCCTCAAAAAGTAGAGGTCGACATCATAGATGACAACTTTATCCTGA----GAACAGGAGTGATGAGTCTGTCGGGAATGTGACTTTTTCATTCGATTATCAAAAGTATGTGACTCTACTTA | 136 | deletion |
| 26 | TGGAAAAAATCTAAAATCTCCTCAAAAAGTAGAGGTCGACATCATAGATGACAACTTTATCCTGA----GAACAGGAGCGATGAGTCTGTCGGGAATGCGACTTTTTCATTCGATTATCAAAAGTATGTGACTCTACTTA | 136 | deletion |
| 27 | TGGAAAAAATCTAAAATCTCCTCAAAAAGTAGAGGTCGACATCATAGATGACAACTTTATCCTGA----GAACAGGGGCGATGAGTCTGTCGGGAATGTGACTTTTTCATTCGATTATCAAAAGTATGTGACTCTACTTA | 136 | deletion |
| 28 | TGGAAAAAATCTAAAATCTCCTCAAAAAGTAGAGGTCGACATCATAGATGACAACTTTATCCTGA----GAACAGGAGCGATGAGTCTGTCAGGAATGTGACTTTTTCATTCGATTATCAAAAGTATGTGACTCTACTTA | 136 | deletion |
| 29 | TGGAAAAAATCTAAAATCTCCTCAAAAAGTAGAGGTCGACATCATAGATGACAACTTTATCCTGAGGTG----AGGAGCGATGAGTCTGTCGAGAATGTGACTTTTTCATTCGATTATCAAAAGTATGTGACTCTACTTA | 136 | deletion |
| 30 | TGGAAAAAATCTAAAATCTCCTCAAAAAGTAGAGGTCGACATCATAGATGATAACTTTATCCTGAGGTG----AGGAGCGATGAGTCTGTCGGGAATGTGACTTTTTCATTCGATTATCAAAAGTATGTGACTCTACTTA | 136 | deletion |
| 31 | TGGAAAAAATCTAAAATCTCCTCAAAAAGTAGAGGTCGACATCATAGATGACAACTTTATCCTGAGGTG----AGGAGCGATGAGTCTGTCGGGAATGTGACTTTTTCATTCGATTATCAAAAGTATGTGACTCTACTTT | 136 | deletion |
| 32 | TGGAAAAAATCTAAAATCTCCTCAAAAAGTAGAGGTCGACATCATAGATGACAACTTTATCCTGA----GAACAGGAACGATGAGTCTGTCGGGAATGTGACTTTTTCATTCGATTATCAAAAGTATGTGACTCTACTTA | 136 | deletion |
| 33 | TGGAAAAAATCTAAAATCTCCTCAAAAAGTAGAGGTCGACATCATAGATGACAACTTTATCCTGAGGTG----AGGAGCGATGAGTCTGTCAGGAATGTGACTTTTTCATTCGATTATCAAAAGTATGTGACTCTACTTA | 136 | deletion |
| 34 | TGGAAAAAATCTAAAATCTCCTCAAAAAGTAGAGGTCGACATCATAGATGACAACTTTATCCTGAGGTG----AGGAGCGATGAGTCTGTCGGGAATGTGACTTTTTCATTTGATTATCAAAAGTATGTGACTCTACTTA | 136 | deletion |
| 35 | TGGAAAAAATCTAAAATCTCCTCAAAAAGTAGAGGTCGACATCATAGATGACAACTTTATCCTGAGGTG----AGGAGCGATGAGTCTGTCGTGAATGTGACTTTTTCATTCGATTATCAAAAGTATGTGACTCTACTTA | 136 | deletion |
| 36 | TGGAAAAAATCTAAAATCTCCTCAAAAAGTAGAGGTCGACATCATAGATGATAACTTTATCCTGA----GAACAGGAGCGATGAGTCTGTCGGGAATGTGACTTTTTCATTCGATTATCAAAAGTATGTGACTCTACTTA | 136 | deletion |
| 37 | TGGAAAAAATCTAAAATCTCCTCAAAAAGTAGAGGTCAACATCATAGATGACAACTTTATCCTGA----GAACAGGAGCGATGAGTCTGTCGGGAATGTGACTTTTTCATTCGATTATCAAAAGTATGTGACTCTACTTA | 136 | deletion |
| 38 | CGGAAAAAATCTAAAATCTCCTCAAAAAGTAGAGGTCGACATCATAGATGACAACTTTATCCTGAGGTG----AGGAGCGATGAGTCTGTCGGGAATGTGACTTTTTCATTCGATTATCAAAAGTATGTGACTCTACTTA | 136 | deletion |
| 39 | AGGAAAAAATCTAAAATCTCCTCAAAAAGTAGAGGTCGACATCATAGATGACAACTTTATCCTGA----GAACAGGAGCGATGAGTCTGTCGGGAATGTGACTTTTTCATTCGATTATCAAAAGTATGTGACTCTACTTA | 136 | deletion |
| 40 | TGGAAAAAATCTAAAATCTCCTCAAAAAGTAGAGGTCGACATCATAGATGACAACTTTATCCTGA----GAACAGGAGCGATGAGTCTGTCGGGAATGTGACTTTTTCATTCGATTATCAAAAGTATGTGGCTCTACTTA | 136 | deletion |
| 41 | TGGAAAAAATCTAAAATCTCCTCAAAAAGTAGAGGTCGACATCATAGATGACAACTTTATCCTGA----GAACAGGAGCGATGAGTCTGTTGGGAATGTGACTTTTTCATTCGATTATCAAAAGTATGTGACTCTACTTA | 136 | deletion |
| 42 | TGGAAAAAATCTAAAATCTCCTCAAAAAGTAGAGGTCGACATCATAGATGACAACTTTATCCTGAGGTG----AGGAGCAATGAGTCTGTCGGGAATGTGACTTTTTCATTCGATTATCAAAAGTATGTGACTCTACTTA | 136 | deletion |
| 43 | TGGAAAAAATCTAAAATCTCCTCAAAAAGTAGAGGTCGACATCATAGATGACAACTTTATCCTGA----GAACAGGAGCGATGAGTCTGTCGAGAATGTGACTTTTTCATTCGATTATCAAAAGTATGTGACTCTACTTA | 136 | deletion |
| 44 | TGGAAAAAATCTAAAATCTCCTCAAAAAGTAGAGGTCGACATCATAGATGACAACTTTATCCTGAGGTG----AGGAGCGATGAGTCTGTCGGGAATGTGATTTTTTCATTCGATTATAAAAAGTATGTGACTCTACTTA | 136 | deletion |
| 45 | TGGAAAAAATCTAAAATCTCCTCAAAAAGTAGAGGTCGACATCATAGATGACAACTTTATCCTGA----GAACAGGAGCGATGAGTCTATCGGGAATGTGACTTTTTCATTCGATTATCAAAAGTATGTGACTCTACTTA | 136 | deletion |
| 46 | TGGAAAAAATCTAAAATCTCCTCAAAAAGTAGAGGTCGACATCATAGATGACAACTTTATCCTGAGGTG----AGGAACGATGAGTCTGTCGGGAATGTGACTTTTTCATTCGATTATCAAAAGTATGTGACTCTACTTA | 136 | deletion |
| 47 | TGGAAAAAATCTAAAATCTCCTCAAAAAGTAGAGGTCGACATCATAGATGACAACTTTATCCTGAGGTG----AGGAGCGATGAGTCTGTCGGGAATGTGATTTTTTCATTCGATTATCAAAAGTATGTGACTCTACTTA | 136 | deletion |
| 48 | TGGAAAAAATCTAAAATCTCCTCAAAAAGTAGAGGTCGACATCATAGATGACAACTTTATCCTGAGGTG----AGGAGCGATGAGTCTGTCGGGAATGTGACTTTTTCATTCGATTATCAAAAGTATGTGGCTCTACTTA | 136 | deletion |
| 49 | TGGAAAAAATCTAAAATCTCCTCAAAAAGTAGAGGTCGACATCATAGATGACAACTTTATCTTGA----GAACAGGAGCGATGAGTCTGTCGGGAATGTGACTTTTTCATTCGATTATCAAAAGTATGTGACTCTACTTA | 136 | deletion |
| 50 | TGAAAAAAATCTAAAATCTCCTCAAAAAGTAGAGGTCGACATCATAGATGACAACTTTATCCTGA----GAACAGGAGCGATGAGTCTGTCGGGAATGTGACTTTTTCATTCGATTATCAAAAGTATGTGACTCTACTTA | 136 | deletion |
| 51 | TGGAAAAAATCTAAAATCTCCTCAAAAAGTAGAGGTCGACATCATAGATGACAACTTTATCCTGA----GAACAGGAGCGATGAGTCTGTCGGGAATGTGACTTTTTCATTCGATTATCAAAAGTATGTGACTCTATTTA | 136 | deletion |
| 52 | TGGAAAAAATCTAAAATCTCCTCAAAAAGTAGAGGTCGACATCATAGATGACAACTTTATCCTGAGGTG----AGGAGCGATGAGTTTGTCGGGAATGTGACTTTTTCATTCGATTATCAAAAGTATGTGACTCTACTTA | 136 | deletion |
| 53 | TGGAAAAAATCTAAAATCTCCTCAAAAAGTAGAGGTCGACATCATAGATGACAATTTTATCCTGAGGTG----AGGAGCGATGAGTCTGTCGGGAATGTGACTTTTTCATTCGATTATCAAAAGTATGTGACTCTACTTA | 136 | deletion |
| 54 | TGGAAAAAATCTAAAATCTCCTCAAAAAGTAGAGGTCGACATCATAGATGACAACTTTATCCTGA----GAACAGGAGCGATGAGTCTGTCGGAAATGTGACTTTTTCATTCGATTATCAAAAGTATGTGACTCTACTTA | 136 | deletion |
| 55 | TGGAAAAAATCTAAAATCTCCTCAAAAAGTAGAGGTCGACATCATAGATGACAACTTTATCCTGAGGTG----AGGAGTGATGAGTCTGTCGGGAATGTGACTTTTTCATTCGATTATCAAAAGTATGTGACTCTACTTA | 136 | deletion |
| 56 | TGGAAAAAATCTAAAATCTCCTCAAAAAGTAGAGGTCGACATCATAGATGACAACTTTATCCTGAGGTG----AGGAGCGATGAGTCTGTCGGGAATGTGACTTTTTCATTCGATTATCAAAAGTATGTGATTCTACTTA | 136 | deletion |
| 57 | TGGAAAAAATCTAAAATCTCCTCAAAAAGTAGAGGTCGACATCATAGATGACAACTTTATCCTGAGGTG----AGGAGCGATGAGTCTGTCGGGAATGCGACTTTTTCATTCGATTATCAAAAGTATGTGACTCTACTTA | 136 | deletion |
| 58 | TGGAAAAAATTTAAAATCTCCTCAAAAAGTAGAGGTCGACATCATAGATGACAACTTTATCCTGAGGTG----AGGAGCGATGAGTCTGTCGGGAATGTGACTTTTTCATTCGATTATCAAAAGTATGTGACTCTACTTA | 136 | deletion |
| 59 | TGGAAAAAATCTAAAATCTCCTCAAAAAGTAGAGATCGACATCATAGATGACAACTTTATCCTGA----GAACAGGAGCGATGAGTCTGTCGGGAATGTGACTTTTTCATTCGATTATCAAAAGTATGTGACTCTACTTA | 136 | deletion |
| 60 | TGGAAAAAATCTAAAATCTCCTCAAAAAGTAGAGGTCGACATCATAGATGACAACTTTATCCTGA----GAACAGGAGCGATGAGTCTGTCGGGAATGTGACTTTTTTATTCGATTATCAAAAGTATGTGACTCTACTTA | 136 | deletion |
| 61 | TGGAAAAAATCTAAAATCTCCTCAAAAAGTAGAGATCGACATCATAGATGACAACTTTATCCTGAGGTG----AGGAGCGATGAGTCTGTCGGGAATGTGACTTTTTCATTCGATTATCAAAAGTATGTGACTCTACTTA | 136 | deletion |
| 62 | TGGAAAAAATCTAAAATCTCCTCAAAAAGTAGAGGTCGACATCATAGATGACAACTTTATCCTGAGGTG----AGGAGCGATGAGTCTGTCGGGAATGTGACTTTTTCATTCGATTATCAAAAGTATGTGACTCTTCTTA | 136 | deletion |
| 63 | TGGAAAAAATCTAAAATCTCCTCAAAAAGTAGAGGTCGACATCATAGATGACAACTTTATCCTGAGGTG----AGGAGCGATGAGTCTGTCGGGAATGTGACTTTTTCATTCGATTATCAAAAGTATGTGACTCTATTTA | 136 | deletion |
| 64 | TGGAAAAAATCTAAAATCTCCTCAAAAAGTAGAGGTCGACATCATAGATGACAACTTTATCCTGAGGTG----AGGAGCGATGAGTCTGTCGGGAATGTGACTTTTTCATTCGATTATCAAAAGTATGTAACTCTACTTA | 136 | deletion |
| 65 | TGGAAAAAATCTAAAATCTCCTCAAAAAGTAGAGGTCGACATCATAGATGACAACTTTATCCTGA----GAACAGGAGCGATGAGTCTGTCGGGAATGTGACTTTTTCATTCGATTATTAAAAGTATGTGACTCTACTTA | 136 | deletion |
| 66 | TGGAAAAAATCTAAAATCTCCTCAAAAAGTAGAGGTCGACATCATAGATGACAACTTTATCCTGA----GAACAGGAGCGATGAGTCTGTCGGGAATGTGATTTTTTCATTCGATTATCAAAAGTATGTGACTCTACTTA | 136 | deletion |
| 67 | TGGAAAAAATCTAAAATCTCCTCAAAAAGTAGAGGTCGACATCATAGATGACAACTTTATCCTGA----GAACAGGAGCGATGAGTCTGTCGGGAATGTGACTTTTTCATTCGATTATCAAAAGTATGTAACTCTACTTA | 136 | deletion |
| 68 | TGGAAAAAATCTAAAATCTCCTCAAAAAGTAGAGGTCGACATCATAGATGGCAACTTTATCCTGAGGTG----AGGAGCGATGAGTCTGTCGGGAATGTGACTTTTTCATTCGATTATCAAAAGTATGTGACTCTACTTA | 136 | deletion |
| 69 | TGGAAAAAATCTAAAATCTCCTCAAAAAGTAGAGGTCGACATCATAGATGACAACTTTATCCTGA----GAACAGGAGCAATGAGTCTGTCGGGAATGTGACTTTTTCATTCGATTATCAAAAGTATGTGACTCTACTTA | 136 | deletion |
| 70 | TGGAAAAAATCTAAAATCTCCTCAAAAAGTAGAGGTCGACATCATAGATGACAACTTTATCTTGAGGTG----AGGAGCGATGAGTCTGTCGGGAATGTGACTTTTTCATTCGATTATCAAAAGTATGTGACTCTACTTA | 136 | deletion |
| 71 | TGGAAAAAATCTAAAATCTCCTCAAAAAGTAGAGGTCGACATCATAGATGACAACTTTATCCTGAGGTG----AGAAGCGATGAGTCTGTCGGGAATGTGACTTTTTCATTCGATTATCAAAAGTATGTGACTCTACTTA | 136 | deletion |
| 72 | TGGAAAAAATCTAAAATCTCCTCAAAAAGTAGAGGTCGACATCATAGATGACAATTTTATCCTGA----GAACAGGAGCGATGAGTCTGTCGGGAATGTGACTTTTTCATTCGATTATCAAAAGTATGTGACTCTACTTA | 136 | deletion |
| 73 | TGGAAAAAATCTAAAATCTCCTCAAAAAGTAGAGGTCGACATCATAGATGACAACTTTATCCTGA----GAACAGGAGCGATGAGTCTGTCGGGAATGTGACTTTTTCATTCGATTATCAAAAGTATATGACTCTACTTA | 136 | deletion |
| 74 | TGGAAAAAATCTAAAATTTCCTCAAAAAGTAGAGGTCGACATCATAGATGACAACTTTATCCTGA----GAACAGGAGCGATGAGTCTGTCGGGAATGTGACTTTTTCATTCGATTATCAAAAGTATGTGACTCTACTTA | 136 | deletion |
| 75 | TGGAAAAAATCTAAAATCTCCTCAAAAAGTAGAGGTCGACATCATAGATGACAACTTTATCCTGA----GAACAGGAGCGATGAGTCTGTCGTGAATGTGACTTTTTCATTCGATTATCAAAAGTATGTGACTCTACTTA | 136 | deletion |
| 76 | TGGAAAAAATCTAAAATCTCCTCAAAAAGTAGAGGTCGACATCATAGATGACAACTTTATCCTGAGGTG----AGGAGCGATGAGTCTGTCGGGAATGTGACTTTTTCATTCGATTATCAAAAGTATGCGACTCTACTTA | 136 | deletion |
| 77 | TGGAAAAAATCTAAAATCTCCTCAAAAAGTAGAGGTCAACATCATAGATGACAACTTTATCCTGAGGTG----AGGAGCGATGAGTCTGTCGGGAATGTGACTTTTTCATTCGATTATCAAAAGTATGTGACTCTACTTA | 136 | deletion |
| 78 | TGGAAAAAATCTAAAATCTCCTC-AAAAGTAGAGGTCGACATCATAGATGACAACTTTATCCTGAGGTG----AGGAGCGATGAGTCTGTCGGGAATGTGACTTTTTCATTCGATTATCAAAAGTATGTGACTCTACTTA | 135 | deletion |
| 79 | TGGAAAAAATTTAAAATCTCCTCAAAAAGTAGAGGTCGACATCATAGATGACAACTTTATCCTGA----GAACAGGAGCGATGAGTCTGTCGGGAATGTGACTTTTTCATTCGATTATCAAAAGTATGTGACTCTACTTA | 136 | deletion |
| 80 | TGGAAAAAATCTAAAATCTCCTCAAAAAGTAGAGGTCGACATCATAGATGACAACTTTATCCTGA----GAACAGGAGCGATGAGTCTGTCGGGAATGTGACTTTTTCATTCGATTATCAAAAGTATGTGACTCTGCTTA | 136 | deletion |
| 81 | TGGAAAAAATCTAAAATCTCCTCAAAAAGTAGAGGTCGACATCATAGATGACAACTTTATCCTGA----GAACAGGAGCGATGAGTCTGTCGGGAATGTGACTTTTTCATTCAATTATCAAAAGTATGTGACTCTACTTA | 136 | deletion |
| 82 | TGGAAAAAATCTAAAATCTCCTCAAAAAGTAGAGGTCGACATCATAGATGACAACTTTATCCTGAGGTG----AGGAGCGATGAGTCTGTCGGGAATGTGACTTTTTCATTCGATTATTAAAAGTATGTGACTCTACTTA | 136 | deletion |

| | | | |
|---|---|---|---|
| 83 | TGGAAAAAATCTAAAATCTCCTCAAAAAGTAGAGGTCGGCATCATAGATGACAACTTTATCCTGAGGTG----AGGAGCGATGAGTCTGTCGGGAATGTGACTTTTTCATTCGATTATCAAAAGTATGTGACTCTACTTA | 136 | deletion |
| 84 | TGGAAAAAATCTAAAATCTCCTCAAAAAATAGAGGTCGACATCATAGATGACAACTTTATCCTGA----GAACAGGAGCGATGAGTCTGTCGGGAATGTGACTTTTTCATTCGATTATCAAAAGTATGTGACTCTACTTA | 136 | deletion |
| 85 | TGGAAAAAATCTAAAATCTCCTCAAAAAGTAGAGGTCGACATCATAGATGACAACTTTATCCTGAGGTG----AGGAGCGATGAGTCTGTCGGGAATGTGACTTTTTTATTCGATTATCAAAAGTATGTGACTCTACTTA | 136 | deletion |
| 86 | TGGAAAAAATCTAAAATCTCCTCAAAAAGTAGAGGTCGACATCATAGATGACAACTTTATCCTAAGGTG----AGGAGCGATGAGTCTGTCGGGAATGTGACTTTTTCATTCGATTATCAAAAGTATGTGACTCTACTTA | 136 | deletion |
| 87 | TAGAAAAAATCTAAAATCTCCTCAAAAAGTAGAGGTCGACATCATAGATGACAACTTTATCCTGAGGTG----AGGAGCGATGAGTCTGTCGGGAATGTGACTTTTTCATTCGATTATCAAAAGTATGTGACTCTACTTA | 136 | deletion |
| 88 | TGGAAAAAATCTAAAATCTTCTCAAAAAGTAGAGGTCGACATCATAGATGACAACTTTATCCTGAGGTG----AGGAGCGATGAGTCTGTCGGGAATGTGACTTTTTCATTCGATTATCAAAAGTATGTGACTCTACTTA | 136 | deletion |
| 89 | TGGAAAAAATCTAAAATCTCCTCAAAAAGTAGAGGTCGACATCATAGATGACAACTTTATTCTGA----GAACAGGAGCGATGAGTCTGTCGGGAATGTGACTTTTTCATTCGATTATCAAAAGTATGTGACTCTACTTA | 136 | deletion |
| 90 | TGGAAAAAATCTAAAATCTCCTCAAAAAGTAGAGGTCGACATCATAGATGACAACTTTATCCTGA----GAACAGGAGCGATGAGTCTGTCGGGAATGTGACTTCTTCATTCGATTATCAAAAGTATGTGACTCTACTTA | 136 | deletion |
| 91 | TGGAAAAAATCTAAAATTTCCTCAAAAAGTAGAGGTCGACATCATAGATGACAACTTTATCCTGAGGTG----AGGAGCGATGAGTCTGTCGGGAATGTGACTTTTTCATTCGATTATCAAAAGTATGTGACTCTACTTA | 136 | deletion |
| 92 | TGGAAAAAATCTAAAATCTCCCCAAAAAGTAGAGGTCGACATCATAGATGACAACTTTATCCTGA----GAACAGGAGCGATGAGTCTGTCGGGAATGTGACTTTTTCATTCGATTATCAAAAGTATGTGACTCTACTTA | 136 | deletion |
| 93 | TGGAAAAAATCTAAAATCTCCTCAAAAAGTAGAGGTCGACATTATAGATGACAACTTTATCCTGAGGTG----AGGAGCGATGAGTCTGTCGGGAATGTGACTTTTTCATTCGATTATCAAAAGTATGTGACTCTACTTA | 136 | deletion |
| 94 | TGGAAAAAATCTAAAATCTCCTCAAAAAGTAGAGGTCGACATCATAGATGACAACTTTATCCCGAGGTG----AGGAGCGATGAGTCTGTCGGGAATGTGACTTTTTCATTCGATTATCAAAAGTATGTGACTCTACTTA | 136 | deletion |
| 95 | TGGAAAAAATCTAAAATCTCCTCAAAAAGTAGAGGTCGACATCATAGATGACAACTTTATCCTGA----GAACAGGAGCGATGAGTCTGTCGGGAATGTGACTTTTTCATTCGATTATCAAAAGTATGTGATTCTACTTA | 136 | deletion |
| 96 | TGGAAAAAATCTAAAATCTCCTCAAAAAGTAGAGGTCGACATCATAGATGACAACTTTATCCTGAGGTG----AGGAGCGATGAGTCTGTCGGGAATGTGTCTTTTTCATTCGATTATCAAAAGTATGTGACTCTACTTA | 136 | deletion |
| 97 | TGGAAAAAATCTAAAATCTCCTCAAAAAGTAGAGGTCGACATCATAGATGACAACTTTATCCTGA----GAACAGGAGCGATGAGTCTGTCGGTAATGTGACTTTTTCATTCGATTATCAAAAGTATGTGACTCTACTTA | 136 | deletion |
| 98 | TGGAAAAAATCTAAAATCTCCTCAAAAAGTAGAGGTCGACATCATAGATGACAACTTTATCCTGA----GAACAGGAGCGATGAGTCTGTCGGGAATGTGACTTTTTCATTCGATTATCAAAAGTATGTGACTCTACTTT | 136 | deletion |
| 99 | TGGAAAAAATCTAAAATCTCTTCAAAAAGTAGAGGTCGACATCATAGATGACAACTTTATCCTGAGGTG----AGGAGCGATGAGTCTGTCGGGAATGTGACTTTTTCATTCGATTATCAAAAGTATGTGACTCTACTTA | 136 | deletion |
| 100 | TGGAAAAAATCTAAAATCTCCTCAAAAAGTAGAGGTCGACATCATAGATGACAACTTTATCCTGA----GAACAGGAGCGATGAGTCTGTCGGGAATGTGACTTTTTCATTCGATTATCAAAAATATGTGACTCTACTTA | 136 | deletion |
| 101 | TGGAAAAAATCTAAAATCTCCTCAAAAAATAGAGGTCGACATCATAGATGACAACTTTATCCTGAGGTG----AGGAGCGATGAGTCTGTCGGGAATGTGACTTTTTCATTCGATTATCAAAAGTATGTGACTCTACTTA | 136 | deletion |
| 102 | TGGAAAAAATCTAAAATCTCCTCAAAAAGTAGAAGTCGACATCATAGATGACAACTTTATCCTGAGGTG----AGGAGCGATGAGTCTGTCGGGAATGTGACTTTTTCATTCGATTATCAAAAGTATGTGACTCTACTTA | 136 | deletion |
| 103 | TGGAAAAAATCTAAAATCTCCTCAAAAAGTAGAGGTCGACATCATAGATGACAACTTTATCCTGA----GAACAGGAGCGATGAGTCTGTCGGGAATGTGACTTTTTCAGTCGATTATCAAAAGTATGTGACTCTACTTA | 136 | deletion |
| 104 | TGGAAAAAATCTAAAATCTCCTCAAAAAGTAGAGGTCGACATCATAGATGACAACTTTATCCTGA----AAACAGGAGCGATGAGTCTGTCGGGAATGTGACTTTTTCATTCGATTATCAAAAGTATGTGACTCTACTTA | 136 | deletion |
| 105 | TGGAAAAAATCTAAAATCTCCTCAAAAAGTAGAGGTCGACATCATAGATGACAACTTTATCCTGAGGTG----AGGAGCGATGAGTCTGTCGGGAATGTGACTTTTTCATTCGATTATCAAAAGTATGTGACTCTACTTG | 136 | deletion |
| 106 | TGGAAAAAATCTAAAATCTCCTCAAAAAGTAGAGGTCGACATCATAGATGACAACTTTATTCTGAGGTG----AGGAGCGATGAGTCTGTCGGGAATGTGACTTTTTCATTCGATTATCAAAAGTATGTGACTCTACTTA | 136 | deletion |
| 107 | TGGAAAAAATCTAAAATCTCCTCAAAAAGTAGAGGTCGACATCATAGATGACAACTTTATCCTGA----GAATAGGAGCGATGAGTCTGTCGGGAATGTGACTTTTTCATTCGATTATCAAAAGTATGTGACTCTACTTA | 136 | deletion |
| 108 | TGGAAAAAATCTAAAATCTCCTCAAAAAGTAGAGGTCGACATCATAGATGACAACTTTATCCTGAGGCG----AGGAGCGATGAGTCTGTCGGGAATGTGACTTTTTCATTCGATTATCAAAAGTATGTGACTCTACTTA | 136 | deletion |
| 109 | TGGAAAAAATCTAAAATCTCCTCAAAAAGTAGAGGTCGACATCATAGATGACAACTTTATCCTGAGGTG----AGGAGCGATGAGTCTGTCGGGAATGTGACTTTTTCATTCGATTATCAAAAATATGTGACTCTACTTA | 136 | deletion |
| 110 | TGGAAAAAATCTAAAATCTCCTCAAAAAGTAGAGGTCGACATCATAGATGACAACTTTATCCTGA----GAACAGGAGCGATGAGTCTGTCGGGAATGTGACTTTTTCATTCGATTATCAAAAGTATGTGACTCTACATA | 136 | deletion |
| 111 | TGGAAAAAATCTAAAATCTCCTCAAAAAGTAGAGGTCGACATCATAGATGACAACTTTATCCTGAGGTG----AGGAGCGATGAGTCTGTCGGGAATGTGACTTTTTCATTCTATTATCAAAAGTATGTGACTCTACTTA | 136 | deletion |
| 112 | TGGAAAAAATCTAAAATCTCCTCAAAAAGTAGAGGCCGACATCATAGATGACAACTTTATCCTGA----GAACAGGAGCGATGAGTCTGTCGGGAATGTGACTTTTTCATTCGATTATCAAAAGTATGTGACTCTACTTA | 136 | deletion |
| 113 | TGGAAAAAATCTAAAATCTCCTCAAAAAGTAGGGGTCGACATCATAGATGACAACTTTATCCTGAGGTG----AGGAGCGATGAGTCTGTCGGGAATGTGACTTTTTCATTCGATTATCAAAAGTATGTGACTCTACTTA | 136 | deletion |
| 114 | TGGAAAAAATCTAAAATCTCCTCAAAAAGTAGAGGTCGACATCATAGATGACAACTTTATCCTAA----GAACAGGAGCGATGAGTCTGTCGGGAATGTGACTTTTTCATTCGATTATCAAAAGTATGTGACTCTACTTA | 136 | deletion |
| 115 | TGGAAAAAATCTAAAATCTCCTCAAAAAGTAGAGGTCGACATCATAGATAACAACTTTATCCTGAGGTG----AGGAGCGATGAGTCTGTCGGGAATGTGACTTTTTCATTCGATTATCAAAAGTATGTGACTCTACTTA | 136 | deletion |
| 116 | AGGAAAAAATCTAAAATCTCCTCAAAAAGTAGAGGTCGACATCATAGATGACAACTTTATCCTGAGGTG----AGGAGCGATGAGTCTGTCGGGAATGTGACTTTTTCATTCGATTATCAAAAGTATGTGACTCTACTTA | 136 | deletion |
| 117 | TGGAAAAAATCTAAAATCTCCTCAAAAAGTAGAGGTCGACATCATAGACGACAACTTTATCCTGAGGTG----AGGAGCGATGAGTCTGTCGGGAATGTGACTTTTTCATTCGATTATCAAAAGTATGTGACTCTACTTA | 136 | deletion |
| 118 | TGGAAAAAATCTAAAATCTCCTC-AAAAGTAGAGGTCGACATCATAGATGACAACTTTATCCTGA----GAACAGGAGCGATGAGTCTGTCGGGAATGTGACTTTTTCATTCGATTATCAAAAGTATGTGACTCTACTTA | 135 | deletion |
| 119 | TGGAAAAAATCTAAAATCTCCTCAAAAAGTAGAGGTCGACATCATAGATGACAACTTTATCCTGA----GAACAGGAGCGATGAGTCTGTCGGGAATGTGACTTTTCCATTCGATTATCAAAAGTATGTGACTCTACTTA | 136 | deletion |
| 120 | TGGAAAAAATCTAAAATCTCCTCAAAAAGTAGAGGTCGACATCATAGATGACAACTTTATCCTGA----GAACAGGAGCGATGAGTCTGTCGGGAATGTGACTTTTTCATTCGATTATCAAAAGTATGAGACTCTACTTA | 136 | deletion |
| 121 | TGGAAAAAATCTAAAATCTCCTCAAAAAGTAGAGGTCGACATCATAGATGACAACTTTATCCTGA----GAACAGGAGCGATGAGTCTGTCGGGAATGTGACTTTTTCATTCGATTATCAAAAGTATGTGACTCTTCTTA | 136 | deletion |
| 122 | TGGAAAAAATCTAAAATCTCCTTAAAAAGTAGAGGTCGACATCATAGATGACAACTTTATCCTGA----GAACAGGAGCGATGAGTCTGTCGGGAATGTGACTTTTTCATTCGATTATCAAAAGTATGTGACTCTACTTA | 136 | deletion |
| 123 | TGGAAAAAATCTAAAATCTCCTCAAAAAGTAGAGGTCGACATCATAGATGACAACTTTATCCTGAGGTG----AGGAGCGATGAGTCTGTCGGGAATGTGAATTTTTCATTCGATTATCAAAAGTATGTGACTCTACTTA | 136 | deletion |
| 124 | TGGAAAAAATCTAAAATCTCCTCAAAAAGTAGAGGTCGACATCATAGATGACAACTTTATCCTGA----GAACAGGAGCGATGAGTCTGTCGGGAATGTGACTTTTTCATACGATTATCAAAAGTATGTGACTCTACTTA | 136 | deletion |
| 125 | TGGAAAAAATCTAAAATCTCCTCAAAAAGTAGAGGTCGGCATCATAGATGACAACTTTATCCTGA----GAACAGGAGCGATGAGTCTGTCGGGAATGTGACTTTTTCATTCGATTATCAAAAGTATGTGACTCTACTTA | 136 | deletion |
| 126 | TGGAAAAAATCTAAAATCTCCTCAAAAAGTAGAGGTCGACATCATAGATGACAACTTTATCCTGAGATG----AGGAGCGATGAGTCTGTCGGGAATGTGACTTTTTCATTCGATTATCAAAAGTATGTGACTCTACTTA | 136 | deletion |
| 127 | TGGAAAAAATCTAAAATCTCCTCAAAAAGTAGAGGTCGACATCATAGATGACAACTTTATCCTGAGGTG----AGGAGCGATGAATCTGTCGGGAATGTGACTTTTTCATTCGATTATCAAAAGTATGTGACTCTACTTA | 136 | deletion |
| 128 | TGGAAAAAATCTAAAATCTCCTCAAAAAGTAGAGGTCGACATCATAGATGACAACTTTATCCTGAGGTG----AGGAGCGATGAGTCTGTCGGGAATGTGACTTTTCCATTCGATTATCAAAAGTATGTGACTCTACTTA | 136 | deletion |
| 129 | TGGAAAAAATCTAAAATCTCCTCAAAAAGTAGAGGTCGACATCATAGATGACAACTTTATCCTGA----GAACAGGAGCGATGAGTCTGTCGGGAATGTGACTTTTTCATTCGATTATCAAAAGTGTGTGACTCTACTTA | 136 | deletion |
| 130 | TGGAAAAAATCTAAAATCTCCTCAAAAAGTAGAGGTCGACATCATAGATGACAACTTTATCCTGA----GAACAGGAGCGATGAGTCTGTCGGGAATGTGACTTTTTCATTCGATTATCAAAAGTATGTGACCCTACTTA | 136 | deletion |
| 131 | TGGAAAAAATCTAAAATCTCCTCAAAAAGTAGAGGTCGACATCATAGATGACAACTTTATCCTGAGGTG----AGGAGCGATGAGTCTGTCGGGAATATGACTTTTTCATTCGATTATCAAAAGTATGTGACTCTACTTA | 136 | deletion |
| 132 | TGGAAAAAATCTAAAATCTCCTCAAAAAGTAGAGGTCGACATCATAGATGACAACTTTATCCTGA----GAACAGGAGCGATGAGTCTGTCGGGAATGTGACTTTTTCATTCTATTATCAAAAGTATGTGACTCTACTTA | 136 | deletion |
| 133 | TGGAAAAAATCTAAAATCTCCTCAAAAAGTAGAGGTCGACATCATAGATGACAACTTTATCCTGA----GAACAGGAGCGATGAGTCTGTCGGGAATGTGACTTTTTCACTCGATTATCAAAAGTATGTGACTCTACTTA | 136 | deletion |
| 134 | TGGAAAAAATCTAAAATCTCCTCAAAAAGTAGAGGTCGACATCGTAGATGACAACTTTATCCTGAGGTG----AGGAGCGATGAGTCTGTCGGGAATGTGACTTTTTCATTCGATTATCAAAAGTATGTGACTCTACTTA | 136 | deletion |
| 135 | TGGAAAAAATCTAAAATCTCCTCAAAAAGTAGAGGTCGACATCATAGATGACAACTTTATCCTGAGGTG----AGGAGCGATGAGTCTATCGGGAATGTGACTTTTTCATTCGATTATCAAAAGTATGTGACTCTACTTA | 136 | deletion |
| 136 | TGGAAAAAATCTAAAATCTCCTCAAAAAGTAGAGGTCGACATCATAGATGACAACTTTATCCCGA----GAACAGGAGCGATGAGTCTGTCGGGAATGTGACTTTTTCATTCGATTATCAAAAGTATGTGACTCTACTTA | 136 | deletion |
| 137 | TGGAAAAAATCTAAAATCTCCTCAAAAAGTAGAAGTCGACATCATAGATGACAACTTTATCCTGA----GAACAGGAGCGATGAGTCTGTCGGGAATGTGACTTTTTCATTCGATTATCAAAAGTATGTGACTCTACTTA | 136 | deletion |
| 138 | TGGAAAAAATCTAAAATCTCCTCAAAAAGTAGAGGTCGACATCATAGATGTCAACTTTATCCTGA----GAACAGGAGCGATGAGTCTGTCGGGAATGTGACTTTTTCATTCGATTATCAAAAGTATGTGACTCTACTTA | 136 | deletion |
| 139 | TGGAAAAAATCTAAAATCTCCTCAAAAAGTAGAGGTCGACATCATAGATGACAACTTTATCCTGAGGTG----AGGAGCGATAAGTCTGTCGGGAATGTGACTTTTTCATTCGATTATCAAAAGTATGTGACTCTACTTA | 136 | deletion |
| 140 | TGAAAAAAATCTAAAATCTCCTCAAAAAGTAGAGGTCGACATCATAGATGACAACTTTATCCTGAGGTG----AGGAGCGATGAGTCTGTCGGGAATGTGACTTTTTCATTCGATTATCAAAAGTATGTGACTCTACTTA | 136 | deletion |
| 141 | TGGAAAAAATCTAAAATCTCCTCAAAAAGTAGAGGTCGACATCATAGATGACAACTTTATCCTGA----GAACAGGAGCGATGAGTCTGTCGGGAATATGACTTTTTCATTCGATTATCAAAAGTATGTGACTCTACTTA | 136 | deletion |
| 142 | TGGAAAAAATCTAAAATCTCCTCAAAAAGTAGAGGTCGACATCATAGATGACAACTTTATCCTGA----GAACAAGAGCGATGAGTCTGTCGGGAATGTGACTTTTTCATTCGATTATCAAAAGTATGTGACTCTACTTA | 136 | deletion |
| 143 | TGGAAAAAATCTAAAATCTCCTCAAAAAGTAGAGGTCGACATCATAGATGACAACTTTATCCTGA----GAACAGGAGCGATGAGTCTGTCGGGAATGTGACTTTTTCATTCGATTATCAAAAGTATGTGACTCTACTTG | 136 | deletion |
| 144 | TGGAAAAAATCTAAAATCTCCCCAAAAAGTAGAGGTCGACATCATAGATGACAACTTTATCCTGAGGTG----AGGAGCGATGAGTCTGTCGGGAATGTGACTTTTTCATTCGATTATCAAAAGTATGTGACTCTACTTA | 136 | deletion |
| 145 | TGGAAAAAATCTAAAATCTCCTCAAAAAGTAGAGGTCGACATCATAGATGACAACTTTATCCTGAGGTG----AGGAGCGATGGGTCTGTCGGGAATGTGACTTTTTCATTCGATTATCAAAAGTATGTGACTCTACTTA | 136 | deletion |
| 146 | TGGAAAAAATCTAAAATCTCCTCAAAAAGTAGAGGACGACATCATAGATGACAACTTTATCCTGA----GAACAGGAGCGATGAGTCTGTCGGGAATGTGACTTTTTCATTCGATTATCAAAAGTATGTGACTCTACTTA | 136 | deletion |
| 147 | TGGAAAAAATCTAAAATCTCCTCAAAAAGTAGAGGTCGATATCATAGATGACAACTTTATCCTGA----GAACAGGAGCGATGAGTCTGTCGGGAATGTGACTTTTTCATTCGATTATCAAAAGTATGTGACTCTACTTA | 136 | deletion |
| 148 | TGGAAAAAATCTAAAATCTCCTCAAAAAGTAGAGGTCGACATCATAGATGACAACTTTATCCTGA----GAACAGGAGCGATGAGTCTGTCGGGAATGTGACTTTTTCATTCGATTATCAAAAGTATGTGACTTTACTTA | 136 | deletion |
| 149 | TGGAAAAAATCTAAAATCTCCTCAAAAAGTAGAGGTCGACATCATAGATGACAACTTTATCCTGAGGTG----AGGAGCGATGAGTCTGTCGGGAATGTGACTTTTTCATTCGATTATCAAAAGTATGTGACTCTACTTC | 136 | deletion |
| 150 | TGGAAAAAATCTAAAATCTCCTCAAAAAGTAGAGGTCGACATCATAGATGACAACTTTATCCTGA----GAACAGGAGCGATGAGTCTGTCGGGAATGTGACTTTTTCATTCGTTTATCAAAAGTATGTGACTCTACTTA | 136 | deletion |
| 151 | TGGAAAAAATCTAAAATCTCCTCAAAAGGTAGAGGTCGACATCATAGATGACAACTTTATCCTGA----GAACAGGAGCGATGAGTCTGTCGGGAATGTGACTTTTTCATTCGATTATCAAAAGTATGTGACTCTACTTA | 136 | deletion |
| 152 | TGGAAAAAATCTAAAATCTCCTCAAAAAGTAGAGGTCGACATCATAGATGACAACTTTATCCTGA----GAACAGGAGCGATGAGTCTGTCGGGAATGTGACCTTTTCATTCGATTATCAAAAGTATGTGACTCTACTTA | 136 | deletion |
| 153 | TGGAAAAAATCTAAAATCTCCTCAAAAAGTAGAGGTCGACATCATAGATGACAACTTTATCCTGAGGTG----AGGAGCGATGAGTCTGTCGGGAATGTGACTTTTTCATTCGATTATCAAAAGTATGTGACTCTACATA | 136 | deletion |
| 154 | TGGAAAAAATCTAAAATCTCCTCAAAAAGTAGAGGTCGACATCATAGATGACAACTTTATCCTGAGGTG----AGGAGCGATGAGTCTGTCGGGAATGTGACTTTTTCATTCGATTATCAAAAGTATGTGTCTCTACTTA | 136 | deletion |
| 155 | TGGAAAAAATCTAAAATCTCCTCAAAAAGTAGAGGTCGATATCATAGATGACAACTTTATCCTGAGGTG----AGGAGCGATGAGTCTGTCGGGAATGTGACTTTTTCATTCGATTATCAAAAGTATGTGACTCTACTTA | 136 | deletion |
| 156 | TGGAAAAAATCTAAAATCTCCTCAAAAAGTAGAGGTCGACATCATAGATGACAACTTTATCCTGA----GAACAGGAGCGATGAGTCTGTCGGGAATGTGACTTTTTCATTCGATTATCAAAGGTATGTGACTCTACTTA | 136 | deletion |
| 157 | TGGAAAAAATCTAAAATCTCCTCAAAAAGTAGAGGTCGACATCATAGATGACAACTTTATCCTGAGGTG----AGGAGCGATGAGTCTGTCGGGAATGTGACTTCTTCATTCGATTATCAAAAGTATGTGACTCTACTTA | 136 | deletion |
| 158 | TGGAAAAAATCTAAAATCTCCTCAAAAAGTAGAGGTCGACATCATAGATGACAAC-TTATCCTGA----GAACAGGAGCGATGAGTCTGTCGGGAATGTGACTTTTTCATTCGATTATCAAAAGTATGTGACTCTACTTA | 135 | deletion |
| 159 | TGGAAAAAATCTAAAATCTCCTCAAAAAGTAGGGGTCGACATCATAGATGACAACTTTATCCTGA----GAACAGGAGCGATGAGTCTGTCGGGAATGTGACTTTTTCATTCGATTATCAAAAGTATGTGACTCTACTTA | 136 | deletion |
| 160 | TGGAAAAAATCTAAAATCTCCTCAAAAAGTAGAGGTCGACATCATAGATGACAACTTTATCCTGAGGTG----AGGAGCGATGAGTCTGTCGGGAATGTGACTTTTTCATACGATTATCAAAAGTATGTGACTCTACTTA | 136 | deletion |
| 161 | TGGAAAAAATCTAAAATCTCCTCAAAAAGTAGAGGTCGACATCATAGATGACAACTTTATCCTGAGGT----TAGGAGCGATGAGTCTGTCGGGAATGTGACTTTTTCATTCGATTATCAAAAGTATGTGACTCTACTTA | 136 | deletion |
| 162 | TGGAAAAAATCTAAAATCTCCTCAAAAAGTAGAGGTCGACATCATAGATGACAACTTTATCCTGA----GAACAGGAGCGATGAGTCTGTCGGGAATGTAACTTTTTCATTCGATTATCAAAAGTATGTGACTCTACTTA | 136 | deletion |
| 163 | TGGAAAAAATCTAAAATCTCCTCAAAAAGTAGAGGTCGACATCATAGATGACAACTTTATCCTGAGGTG----AGGAGCGATGAGTCTGTCGGGAATGTAACTTTTTCATTCGATTATCAAAAGTATGTGACTCTACTTA | 136 | deletion |
| 164 | TGGAAAAAATCTAAAATCTCCTCAAAAAGTAGAGGTCGACATCATAGATGACAACTTTATCCTGA----GAACAGGAGCGATGAGTCTGTCGGGAATGTGACTTTTTCATTCGATTATCAAAAGTACGTGACTCTACTTA | 136 | deletion |
| 165 | TGGAAAAAATCTAAAATCTCCTCAAAAAGTAGAGGTCGACATCATAGATGACAACTTTATCCTGAGGTG----AGGAGCGATGAGTCTGTCGGGAATGTGACTTTTTCATTCGATTAACAAAAGTATGTGACTCTACTTA | 136 | deletion |
| 166 | TGGAAAAAATCTAAAATCTCCTCAAAAAGTAGAGGTCGACATCATAGATGACAACTTTATCCTGAGGTG----AGGAGCGATGAGTCTGTCGGGAATGAGACTTTTTCATTCGATTATCAAAAGTATGTGACTCTACTTA | 136 | deletion |
| 167 | TGGAAAAAATCTAAAATCTCCTCAAAAAGTAGAGGTCGACATCATAGATGTCAACTTTATCCTGAGGTG----AGGAGCGATGAGTCTGTCGGGAATGTGACTTTTTCATTCGATTATCAAAAGTATGTGACTCTACTTA | 136 | deletion |
| 168 | TGGAAAAAATCTAAAATCTCCTCAAAAAGTAGAGGTCGACATCATAGATGACAACTTTATCCTGAGGTG----AGGAGCGATGAGTCTGTCGGGAATGTGACTTTGTCATTCGATTATCAAAAGTATGTGACTCTACTTA | 136 | deletion |

| | | | |
|---|---|---|---|
| 169 | TGGAAAAAATCTAAAATCTCCTCAAAAAGTAGAGGTCGACATCATAGATGACAACTTTATCCTG----GGAACAGGAGCGATGAGTCTGTCGGGAATGTGACTTTTTCATTCGATTATCAAAAGTATGTGACTCTACTTA | 136 | deletion |
| 170 | GGGAAAAAATCTAAAATCTCCTCAAAAAGTAGAGGTCGACATCATAGATGACAACTTTATCCTGAGGTG----AGGAGCGATGAGTCTGTCGGGAATGTGACTTTTTCATTCGATTATCAAAAGTATGTGACTCTACTTA | 136 | deletion |
| 171 | TGGAAAAAATCTAAAATCTCCTCAAAAAGTAGAGGTCGACATCATAGATGACAACTTTATCCTGAGG----ACAGGAGCGATGAGTCTGTCGGGAATGTGACTTTTTCATTCGATTATCAAAAGTATGTGACTCTACTTA | 136 | deletion |
| 172 | TGGAAAAAATCTAAAATCTCCTCAAAAAGTAGAGGTCGACATCATAGATGACAACTTTATCCTGA----GAACAGGAGCGATGAGTCTGTCGGGAATGTGACTTTTTCATTCGATTATCAAAAGTATGTGTCTCTACTTA | 136 | deletion |
| 173 | TGGAAAAAATCTAAATTCTCCTCAAAAAGTAGAGGTCGACATCATAGATGACAACTTTATCCTGA----GAACAGGAGCGATGAGTCTGTCGGGAATGTGACTTTTTCATTCGATTATCAAAAGTATGTGACTCTACTTA | 136 | deletion |
| 174 | TGGAAAAAATCTAAAATCTCCTCAAAAAGTAGAGGTCGACATCATAGATGACAACTTTATCCTGAGGTG----AGGAGCGATGAGTCTGTCGGGAATGTGACTTTTTCATTAGATTATCAAAAGTATGTGACTCTACTTA | 136 | deletion |
| 175 | TGGAAAAAATCTAAAATCTCCTCAAAAAGTAGAGGTCGACATCATAGATGACAACTTTATCCTGA----GAACAGGAGCTATGAGTCTGTCGGGAATGTGACTTTTTCATTCGATTATCAAAAGTATGTGACTCTACTTA | 136 | deletion |
| 176 | TGGAAAAAATCTAAAATCTCCTCAAAAAGTAGAGGTCGACATCATAGATGACAACTTTATCCTGAGGTG----AGGAGCGATGAGTCTGTCGGAAATGTGACTTTTTCATTCGATTATCAAAAGTATGTGACTCTACTTA | 136 | deletion |
| 177 | TGGAAAAAATCTAAAATCTCCTCAAAAAGTAGAGGTCGACATCATAGATGACAACTTTATCCTGAGGTG----AGGAGCGATGAGTCTGTCGGGAATGTGACTTTTTCATTCAATTATCAAAAGTATGTGACTCTACTTA | 136 | deletion |
| 178 | TGGAAAAAATCTAAAATCTCCTCAAAAAGTAGAGGTCGACATCATAGATGACAACTTTATCCTGA----GAACAGGAGCGATGAGTCTGTCGGGAATGTGACTTTTTCAATCGATTATCAAAAGTATGTGACTCTACTTA | 136 | deletion |
| 179 | TGGAAAAAATCTAAAATCTCCTCAAAAAGTAGAGGTCGACATCATAGATGACAACTTTATCCTGA----GAACGGGAGCGATGAGTCTGTCGGGAATGTGACTTTTTCATTCGATTATCAAAAGTATGTGACTCTACTTA | 136 | deletion |
| 180 | TGGAAAAAATCTAAAATCTCCTCAAAAAGTAGAGGTCGACATCATAGATGACAACTTTATCCTGA----GAACAGGAGCGATGAGTTTGTCGGGAATGTGACTTTTTCATTCGATTATCAAAAGTATGTGACTCTACTTA | 136 | deletion |
| 181 | TGGAAAAAATCTAAAATCTCCTCAAAAAGTAGAGGTCGACATCATAGATGACAACCTTATCCTGAGGTG----AGGAGCGATGAGTCTGTCGGGAATGTGACTTTTTCATTCGATTATCAAAAGTATGTGACTCTACTTA | 136 | deletion |
| 182 | TGGAAAAAATCTAAAATCTCCTCAAAAAGTAGAGGTCGACATCATAGATGACAACTTTATCCTGA----GAACAGGAGCGATGAATCTGTCGGGAATGTGACTTTTTCATTCGATTATCAAAAGTATGTGACTCTACTTA | 136 | deletion |
| 183 | TGGAAAAAATCTAAAATCTCCTCAAAAAGTAGAGGTCGACATCATAGATGACAACTTTATCCTGAGGTG----AGGAGCGATGAGTCTGTCGGGAATGTGACTTTTTCATTCGATTATCAAAAGTATATGACTCTACTTA | 136 | deletion |
| 184 | TGGAAAAAATCTAAAATCTCCTCAAAAGGTAGAGGTCGACATCATAGATGACAACTTTATCCTGAGGTG----AGGAGCGATGAGTCTGTCGGGAATGTGACTTTTTCATTCGATTATCAAAAGTATGTGACTCTACTTA | 136 | deletion |
| 185 | TGGAAAAAATCTAAAATCTCCTCAAAAAGTAGAGGTCGACATCATAGATGACAACTTTATCCTGAGGTG----AGGAGCGATGAGTCTGTCGGGGATGTGACTTTTTCATTCGATTATCAAAAGTATGTGACTCTACTTA | 136 | deletion |
| 186 | TGGAAAAAATCTAAAATCTCCTCAAAAAGTAGAGGTCGACATCATAGATGACAACTTTATCCTGAGGTG----AGGAGCGATGAGTCTGTCGGGAATGTGAC-TTTTCATTCGATTATCAAAAGTATGTGACTCTACTTA | 135 | deletion |
| 187 | TGGAAAAAATCTAAAATCTCCACAAAAAGTAGAGGTCGACATCATAGATGACAACTTTATCCTGAGGTG----AGGAGCGATGAGTCTGTCGGGAATGTGACTTTTTCATTCGATTATCAAAAGTATGTGACTCTACTTA | 136 | deletion |
| 188 | TAGAAAAAATCTAAAATCTCCTCAAAAAGTAGAGGTCGACATCATAGATGACAACTTTATCCTGA----GAACAGGAGCGATGAGTCTGTCGGGAATGTGACTTTTTCATTCGATTATCAAAAGTATGTGACTCTACTTA | 136 | deletion |
| 189 | TGGAAAAAATCTAAAATCTCCTCAAAAAGTAGAGGTCGACATCATAGATGACAACTTTATCCTGA----GAACAGAAGCGATGAGTCTGTCGGGAATGTGACTTTTTCATTCGATTATCAAAAGTATGTGACTCTACTTA | 136 | deletion |
| 190 | TGGGAAAAATCTAAAATCTCCTCAAAAAGTAGAGGTCGACATCATAGATGACAACTTTATCCTGAGGTG----AGGAGCGATGAGTCTGTCGGGAATGTGACTTTTTCATTCGATTATCAAAAGTATGTGACTCTACTTA | 136 | deletion |
| 191 | TGGAAAAAATCTAAAATCTCCTCAAAAAGTAGAGGTCGACATCATAGATGACAACTTTATCCTGA----GAACAGGAGCGATGAGTCTGTCGGGAATGTGTCTTTTTCATTCGATTATCAAAAGTATGTGACTCTACTTA | 136 | deletion |
| 192 | TGGAAAAAATCTAAAATCTCCTCAAAAAGTAGAGGTCGACATCGTAGATGACAACTTTATCCTGA----GAACAGGAGCGATGAGTCTGTCGGGAATGTGACTTTTTCATTCGATTATCAAAAGTATGTGACTCTACTTA | 136 | deletion |
| 193 | TGGAAAAAATCTAAAATCTCCTCAAAAAGTAGAGGTCGACATCATAGATGACAACTTTATCCTGA----GAACAGGAGCGATGAGTCTGTCGGGAATGTGACTTTTTCATTCGATTATCAAAAGTATGTTACTCTACTTA | 136 | deletion |
| 194 | TGGAAAAAATCTAAAATCTCCTCAAAAAGTAGAGGTCGACATCATAGATGACAACTTTATCCTGA----GAACAGGAGCGATGAGTCTGTCTGGAATGTGACTTTTTCATTCGATTATCAAAAGTATGTGACTCTACTTA | 136 | deletion |
| 195 | TGGAAAAAATCTAAAATCTCCTCAAAAAGTAGAGGTCGACATCATAGATGACAACTTTATCCTGAGGTG----AGGGGCGATGAGTCTGTCGGGAATGTGACTTTTTCATTCGATTATCAAAAGTATGTGACTCTACTTA | 136 | deletion |
| 196 | TGGAAAAAATCTAAAATCTCCTCAAAAAGTGGAGGTCGACATCATAGATGACAACTTTATCCTGA----GAACAGGAGCGATGAGTCTGTCGGGAATGTGACTTTTTCATTCGATTATCAAAAGTATGTGACTCTACTTA | 136 | deletion |
| 197 | TGGAAAAAATCTAAAATCTACTCAAAAAGTAGAGGTCGACATCATAGATGACAACTTTATCCTGA----GAACAGGAGCGATGAGTCTGTCGGGAATGTGACTTTTTCATTCGATTATCAAAAGTATGTGACTCTACTTA | 136 | deletion |
| 198 | TGGAAAAAATCTAAAATCTCCTCAAAAAGTAAAGGTCGACATCATAGATGACAACTTTATCCTGA----GAACAGGAGCGATGAGTCTGTCGGGAATGTGACTTTTTCATTCGATTATCAAAAGTATGTGACTCTACTTA | 136 | deletion |
| 199 | TGGAAAAAATCTAAAATCTCCTCAAAAAGTAGAGGTCGACATCATAGATGACAACTTTATCCTGA----GAACAGGAGCGATGAGTCTGTCGGGAATGTGACTTTGTCATTCGATTATCAAAAGTATGTGACTCTACTTA | 136 | deletion |
| 200 | TGGAAAAAATCTAAAATCTCCTCAAAAAGTAGAGGTCGACATCATAAATGACAACTTTATCCTGAGGTG----AGGAGCGATGAGTCTGTCGGGAATGTGACTTTTTCATTCGATTATCAAAAGTATGTGACTCTACTTA | 136 | deletion |
| 201 | TGGAAAAAATCTAAAATCTCCTCAAAAAGTAGAGGTCGACATCATAGATGACAACTTTATCCTGAGGT----AAGGAGCGATGAGTCTGTCGGGAATGTGACTTTTTCATTCGATTATCAAAAGTATGTGACTCTACTTA | 136 | deletion |
| 202 | TGGAAAAAATCTAAAATCTCCTCAAAAAGTAGAGGTCGACATCATAGATGACAACTTTATCCTGAGGTG----AGGAGCGATGAGTCTGTCGGGAATGTTACTTTTTCATTCGATTATCAAAAGTATGTGACTCTACTTA | 136 | deletion |
| 203 | TGGAAAAAATCTAAAATCTCCTCAAAAAGTAGAGGTCGACATCATAGATGACAACTTTATCCTGAGGTG----AGGAGCGATGAGTCTGTCGGGAATGTGACTTTTTCATTCGATTATCAAAAGTATGTGACTCTACCTA | 136 | deletion |
| 204 | TGGAAAAAATCTAAAATCTCCTCAAAAAGTAGAGGTCGACATCATAGATGACAACTTTATCCTGA----GAACAGGAGCGATGAGTCTGTCGGGAATGTGACTTTTTCATTCGATTATCAAAAGTATGTGACTCTACTTC | 136 | deletion |
| 205 | TGGAAAAAATCTAAAATCTCCTCAAAAAGTAGAGGTCGACATCATAGATGACAACTTTATCCTGA----GAACAGGAGCGATAAGTCTGTCGGGAATGTGACTTTTTCATTCGATTATCAAAAGTATGTGACTCTACTTA | 136 | deletion |
| 206 | TGGAAAAAATCTAAAATCTCCTCAAAAAGTAGAGGTCGACATCATTGATGACAACTTTATCCTGAGGTG----AGGAGCGATGAGTCTGTCGGGAATGTGACTTTTTCATTCGATTATCAAAAGTATGTGACTCTACTTA | 136 | deletion |
| 207 | TGGAAAAAATCTAAAATCTCCTCAAAAAGTAGAGGTCGACATCATAGATGACAACTTTATCCTGAGGTG----AGGAGCGATGAGTCTGTCGGGAATGTGACTTTTTCATTCGATTATCAAAAGTATGGGACTCTACTTA | 136 | deletion |
| 208 | TGGAAAAAATCTAAAATCTCCTCAAAAAGTAGAGGTCGACATCATAGATGACAACTTCATCCTGA----GAACAGGAGCGATGAGTCTGTCGGGAATGTGACTTTTTCATTCGATTATCAAAAGTATGTGACTCTACTTA | 136 | deletion |
| 209 | TGGAAAAAATCTAAAATCTCCTCAAAAAGTAGAGGTCGACATCATAGATGACAACTTTATCCTGAGGTG----AGGAGCGATGAGTCTGTCGGGAACGTGACTTTTTCATTCGATTATCAAAAGTATGTGACTCTACTTA | 136 | deletion |
| 210 | TGGAAAAAATCTAAAATCTCCTCAAAAAGTAAAGGTCGACATCATAGATGACAACTTTATCCTGAGGTG----AGGAGCGATGAGTCTGTCGGGAATGTGACTTTTTCATTCGATTATCAAAAGTATGTGACTCTACTTA | 136 | deletion |
| 211 | TGGAAAAAATCTAAAATCTCCTCAAAAAGTAGAGGTCGACATCATAGATGACAACTTTATCCTGA----GAACAGGAGCGATTAGTCTGTCGGGAATGTGACTTTTTCATTCGATTATCAAAAGTATGTGACTCTACTTA | 136 | deletion |
| 212 | TGGAAAAAATCTAAAATCTCCTCAAAAAGTAGAGGTCGACATCATAGATGACAACTTTATCCTGA----GAACAGGAGCGATGAGTCTGTCGGGAATGTGACTTTTTCATTCGATTATAAAAAGTATGTGACTCTCCTTA | 136 | deletion |
| 213 | TGGAAAAAATCTAAAATCTCCTCAAAAAGTAGAGGTCGACATCATAGATGACAACTTTATCCTGA----GAACAGGAGCGATGAGTCTGCCGGGAATGTGACTTTTTCATTCGATTATCAAAAGTATGTGACTCTACTTA | 136 | deletion |
| 214 | TGGAAAAAATCTAAAATCTCCTCAAAAAGTAGAGGTCGACATCATAGATGACAACTTTATCCTGAGGTG----AGGAGCGATGAGTCTGTCGGGAATGTGACTTTTTCACTCGATTATCAAAAGTATGTGACTCTACTTA | 136 | deletion |
| 215 | TGGAAAAAATCTAAAATCTCCTCAAAAAGTAGAGGTCGACATCATAGATGACAACCTTATCCTGA----GAACAGGAGCGATGAGTCTGTCGGGAATGTGACTTTTTCATTCGATTATCAAAAGTATGTGACTCTACTTA | 136 | deletion |
| 216 | TGGAAAAAATCTAAAATCTCCTCAAAAAGTAGAGGTCGACATCATAGATGACAACTTTATCCTGAGGTG----AGGAGCGATGAGTCTGTCGGGAATGTGACTTTTTCATGCGATTATCAAAAGTATGTGACTCTACTTA | 136 | deletion |
| 217 | TGGAAAAAATCTAAAATCTCCTCAAAAAGCAGAGGTCGACATCATAGATGACAACTTTATCCTGAGGTG----AGGAGCGATGAGTCTGTCGGGAATGTGACTTTTTCATTCGATTATCAAAAGTATGTGACTCTACTTA | 136 | deletion |
| 218 | TGGAAAAAATCTAAAATCTCCTCAAAAAGTAGAGGTCGACATCATAGATGACGACTTTATCCTGA----GAACAGGAGCGATGAGTCTGTCGGGAATGTGACTTTTTCATTCGATTATCAAAAGTATGTGACTCTACTTA | 136 | deletion |
| 219 | TGGAAAAAATCTAAAATCTCCTCAAAAAGTAGAGGTCGACATCATAGATGACAACTTTATCCTGA----GAACAGGAGCGATGAGTCTGTCGGGAATGTGACTTTTTAATTCGATTATCAAAAGTATGTGACTCTACTTA | 136 | deletion |
| 220 | TGGAAAAAATCTAAAATCTCCTCAAAAAGTAGAGGTCTACATCATAGATGACAACTTTATCCTGAGGTG----AGGAGCGATGAGTCTGTCGGGAATGTGACTTTTTCATTCGATTATCAAAAGTATGTGACTCTACTTA | 136 | deletion |
| 221 | TGGAAAAAATCTAAAATCTCCTCAAAAAGTAGAGGTCGACATCATAGATGACAACTTTATCCTGAGGTG----AGGAGCAATGAGTCTGTCGGGAATGTGACTTTTTCATTCGATTATCAAAAGTATGTGACTCTACTGA | 136 | deletion |
| 222 | TGGAAAAAATCTAAAATCTCCTCAAAAAGTAGAGGTCGACATCATAGAAGACAACTTTATCCTGA----GAACAGGAGCGATGAGTCTGTCGGGAATGTGACTTTTTCATTCGATTATCAAAAGTATGTGACTCTACTTA | 136 | deletion |
| 223 | TGGAAAAAATCTAAAATCTCCTCAAAAAGTAGAGGTCGACATCATAGATGACAACTTTATCCTGAGGTG----TGGAGCGATGAGTCTGTCGGGAATGTGACTTTTTCATTCGATTATCAAAAGTATGTGACTCTACTTA | 136 | deletion |
| 224 | TGGAAAAAATCTAAAATCTCCTCAAAAAGTAGAGGTCGACATCATAGATGACAACTTTATCCTGA----GAACAGGAGCGATGAGTCTGTAGGGAATGTGACTTTTTCATTCGATTATCAAAAGTATGTGACTCTACTTA | 136 | deletion |
| 225 | TGGAAAAAATCTAAAATCTCCTCAAAAAGTAGAGGTCGACATCATAGATGACAACTTTATCCTGA----GAACAGGAGCGATGAGTCCGTCGGGAATGTGACTTTTTCATTCGATTATCAAAAGTATGTGACTCTACTTA | 136 | deletion |
| 226 | TGGAAAAAATCTAAAATCTCCTCAAAAAGTAGAGGTCGACATCATAGATGACAACTTTATCCTGAGGTG----AGGAGCGATGAGTCTGTCGGGAATGTGACTTTTTCAGTCGATTATCAAAAGTATGTGACTCTACTTA | 136 | deletion |
| 227 | TGGAAAAAATCTAAAATCTCCTCAAAAAGTAGAGGTCGACATCATAGATGACAACTTTATCCTGAGGTG----AGGAGCGATGAGTCTGTCGGGAATGTGACTTTTTCATTCGATTATCAAAAGTGTGTGACTCTACTTA | 136 | deletion |
| 228 | TGGAAAAAATCTAAAATCTCCTCAAAAAGTATAGGTCGACATCATAGATGACAACTTTATCCTGA----GAACAGGAGCGATGAGTCTGTCGGGAATGTGACTTTTTCATTCGATTATCAAAAGTATGTGACTCTACTTA | 136 | deletion |
| 229 | TGGAAAAAATCTAAAATCTCCTCAAAAAGTAGAGGTCGACATCATAGATGACAACTTTATCCTGA----GATCAGGAGCGATGAGTCTGTCGGGAATGTGACTTTTTCATTCGATTATCAAAAGTATGTGACTCTACTTA | 136 | deletion |
| 230 | TGGAAAAAATCTAAAATCTCCTCAAAAAGTAGAGGTCGACATCATAGATAACAACTTTATCCTGA----GAACAGGAGCGATGAGTCTGTCGGGAATGTGACTTTTTCATTCGATTATCAAAAGTATGTGACTCTACTTA | 136 | deletion |
| 231 | TGGAAAAAATCTAAAATCTCCTCAAAAAGTAGAGGTCGACATCATAGATGACAACTTTATCCTGA----GAACAGGAGCGATGAGTCTGTCGGGAATGTGAC-TTTTCATTCGATTATCAAAAGTATGTGACTCTACTTA | 135 | deletion |
| 232 | TGGAAAAAATCTAAAATCTCCTCAAAAAGTAGAGGTCGACATCATAGATGACAACTTTATCCTGAGGTG----AGGAGCGATGAGTCTGTCGGGAATGTGACTTTTTCATTCGATTATCAAAGGTATGTGACTCTACTTA | 136 | deletion |
| 233 | TGGAAAAAATCTAAAATCTCCTCAAAAAGTAGAGGTCGACATCATAGATGACAACTTTATCCTGA----GAACAGGAGCGATGAGTCTGTCGGGAATGTGACTTTTTCATTCGATTATCAAAAGTATGTGACTCTACCTA | 136 | deletion |
| 234 | GGGAAAAAATCTAAAATCTCCTCAAAAAGTAGAGGTCGACATCATAGATGACAACTTTATCCTGA----GAACAGGAGCGATGAGTCTGTCGGGAATGTGACTTTTTCATTCGATTATCAAAAGTATGTGACTCTACTTA | 136 | deletion |
| 235 | TGGAAAAAATCTAAAATCTCCTCAAAAAGTAGAGGTCGACATCATAGATGACAACTTTATCCTGA----GAACAGGAGCGATGAGTCTGTCGGGAATGTGATTTTTTCATTCGATTATAAAAAGTATGTGACTCTACTTA | 136 | deletion |
| 236 | TGGAAAAAATCTAAAATCTCCTCAAAAAGTAGAGGTCGACATCATAGATGACAACTTTGTCCTGAGGTG----AGGAGCGATGAGTCTGTCGGGAATGTGACTTTTTCATTCGATTATCAAAAGTATGTGACTCTACTTA | 136 | deletion |
| 237 | TGGAAAAAATCTAAAATCTCCTCAAAAAGTAGAGGTCGACATCATAGATGACAACTTTATCCTGA----GAACAGGAGCGATGAGTCTGTCGGGAATGTGACTTTTTCATTCGATTACCAAAAGTATGTGACTCTACTTA | 136 | deletion |
| 238 | TGGAAAAAATCTAAAATCTCCTCAAAAAGTAGAGGTCGACATCATAGATGACAACTTTATCCTGA----GAACAGGAGCGATGAGTCTGTCGGGAATGTGACTTTTTCATTCGATTATAAAAAGTATGTGACTCTACTGA | 136 | deletion |
| 239 | TGGAAAAAATCTAAAATCTCCTCAAAAAGTAGAGGTCGACATCATAGATGACAACTTTATCCTGA----GAACAGGAGCGATGAGTCTGTCGGGAATGTGACTTTTTCATTCGATTATCAAAAGTATGTGACTCTACGTA | 136 | deletion |
| 240 | TGGAAAAAATCTAAAATCTCCTCAAAAAGTAGAGGTCGACATCATAGACGACAACTTTATCCTGA----GAACAGGAGCGATGAGTCTGTCGGGAATGTGACTTTTTCATTCGATTATCAAAAGTATGTGACTCTACTTA | 136 | deletion |
| 241 | TGGAAAAAATCTAAAATCTCCTCAAAAAGTAGAGGTCGACATCATAGATGACAACTTTATCCTGAGGTG----AGGAGCGATGAGTCTGTCGGGAATGTGACTTTTTCATTCGATTATCAAAAGGATGTGACTCTACTTA | 136 | deletion |
| 242 | TGGAAAAAATCTAAAATCTCCTCAAAAAGTAGAGGTCGACATCATAGATGACAACTTTATCCTGAGGTG----AGGAGCGATGAGTCTGTCGGGAATGTGACTTTTTCATTCGATTATCGAAAGTATGTGACTCTACTTA | 136 | deletion |
| 243 | TGGAAAAAATCTAAAATCTCCTCAAAAAGTAGAGGTCGACATCATAGATGACAACTTAATCCTGA----GAACAGGAGCGATGAGTCTGTCGGGAATGTGACTTTTTCATTCGATTATCAAAAGTATGTGACTCTACTTA | 136 | deletion |
| 244 | TGGAAAAAATCTAAAATCTCCTCAAAAAGTAGAGGTCGACATCATAGATGACAACTTTATCCTGAGGTG----AGGAGCGATGAGTCTGTCGGGAATGTGACTTTTTCATTCGATTATCAAAAGTATGTGACCCTACTTA | 136 | deletion |
| 245 | TGGAAAAAATCTAAAATCTCCTCAAAAAGTGGAGGTCGACATCATAGATGACAACTTTATCCTGAGGTG----AGGAGCGATGAGTCTGTCGGGAATGTGACTTTTTCATTCGATTATCAAAAGTATGTGACTCTACTTA | 136 | deletion |
| 246 | TGGAAAAAATCTAGAATCTCCTCAAAAAGTAGAGGTCGACATCATAGATGACAACTTTATCCTGA----GAACAGGAGCGATGAGTCTGTCGGGAATGTGACTTTTTCATTCGATTATCAAAAGTATGTGACTCTACTTA | 136 | deletion |
| 247 | TGGAAAAAATCTAAAATCTCCTCAAAAAGTAGAGGTCGACATCATAGATGACAACTTTATCCTGA----GAACAGGAGCGATGAGTCTGTCGGGAATGTGACTTTTTCATTCGATTATCAAAAGTATGTGAATCTACTTA | 136 | deletion |
| 248 | TGGAAAAAATCTAAAATCTCCTCAAAAAGTAGAGGTCGACATCATAGATGACAACTTTATCCTGAGGTG----AGGAGCGATGAGTCTGTCGGGAATGTGACTTTTTCATTCGATTATCAAAAGTATGTGAATCTACTTA | 136 | deletion |
| 249 | TGGAAAAAATCTAAAATCTCCTCAAAAAGTAGAGGTCGACATCATAGATGACAACTTTATCCTGA----GAACAGGAGCGATGAGTCTGACGGGAATGTGACTTTTTCATTCGATTATCAAAAGTATGTGACTCTACTTA | 136 | deletion |
| 250 | TGGAAAAAATCTAAAATCTCCTCAAAAAGTAGAGGTCGACATCATAGATGACAACTTTATCCTGAGGTG----AGGAGCGATGAGTCTGTCGGGAATGTGACTTTTTCAATCGATTATCAAAAGTATGTGACTCTACTTA | 136 | deletion |
| 251 | TGGAAAAAATCTAAAATCTCCTCAAAAAGTAGAGGTCGACATCATAGATGACAACTTTATCCTGA----GAACAGGAGCGATGAGTCTGTCGGGTATGTGACTTTTTCATTCGATTATCAAAAGTATGTGACTCTACTTA | 136 | deletion |
| 252 | TGGAAAAAATCTAAAATCTCCTCAAAAAGTAGAGGTCGACTTCATAGATGACAACTTTATCCTGAGGTG----AGGAGCGATGAGTCTGTCGGGAATGTGACTTTTTCATTCGATTATCAAAAGTATGTGACTCTACTTA | 136 | deletion |
| 253 | TGGAAAAATTCTAAAATCTCCTCAAAAAGTAGAGGTCGACATCATAGATGACAACTTTATCCTGA----GAACAGGAGCGATGAGTCTGTCGGGAATGTGACTTTTTCATTCGATTATCAAAAGTATGTGACTCTACTTA | 136 | deletion |
| 254 | TGGAAAAAATCTAAGATCTCCTCAAAAAGTAGAGGTCGACATCATAGATGACAACTTTATCCTGA----GAACAGGAGCGATGAGTCTGTCGGGAATGTGACTTTTTCATTCGATTATCAAAAGTATGTGACTCTACTTA | 136 | deletion |

| | | | |
|---|---|---|---|
| 255 | TGGAAAAAATCTAAAATCTCCTCAAAAAGTAGAGGTCGACATCATAGATGACAACTTTATCCTGA----GAACAGGAGCGATGTGTCTGTCGGGAATGTGACTTTTTCATTCGATTATCAAAAGTATGTGACTCTACTTA | 136 | deletion |
| 256 | TGGAAAAAATCTAAAATCTCCTCAAAAAGTAGAGGTCGACAACATAGATGACAACTTTATCCTGA----GAACAGGAGCGATGAGTCTGTCGGGAATGTGACTTTTTCATTCGATTATCAAAAGTATGTGACTCTACTTA | 136 | deletion |
| 257 | TGGAAAAAATCTAAAATCTCCTCAAAAAGTAGAGGTCGACATCATAGATGACAACTTTATCCTGAGGTG----AGGAGCGATGAGTCTGCCGGGAATGTGACTTTTTCATTCGATTATCAAAAGTATGTGACTCTACTTA | 136 | deletion |
| 258 | TGGAAAAAATCTAAAATCTCCTCAAAAAGTAGAGGTCGACATCATAGATGACAACTTTATCCTGA----GAACAGGAGCGATGAGTCTGTCGGGAATGTGACATTTTCATTCGATTATCAAAAGTATGTGACTCTACTTA | 136 | deletion |
| 259 | TGGAAAAAATCTAAAATCTCCTCAAAAAGTAGAGGTCGACATCATAGATGACAACTTTATCCTGAGGTG----AGGAGCGATGAGTCTGTCGGGAATGTGACCTTTTCATTCGATTATCAAAAGTATGTGACTCTACTTA | 136 | deletion |
| 260 | TGGAAAAAATCTAAAATCTCCTCAAAAAGTAGTGGTCGACATCATAGATGACAACTTTATCCTGAGGTG----AGGAGCGATGAGTCTGTCGGGAATGTGACTTTTTCATTCGATTATCAAAAGTATGTGACTCTACTTA | 136 | deletion |
| 261 | TGGAAAAAATCTAAAATCTCCTCGAAAAGTAGAGGTCGACATCATAGATGACAACTTTATCCTGAGGTG----AGGAGCGATGAGTCTGTCGGGAATGTGACTTTTTCATTCGATTATCAAAAGTATGTGACTCTACTTA | 136 | deletion |
| 262 | TGGAAAAAATCTAAAATCTCCTCAAAAAGTTGAGGTCGACATCATAGATGACAACTTTATCCTGAGGTG----AGGAGCGATGAGTCTGTCGGGAATGTGACTTTTTCATTCGATTATCAAAAGTATGTGACTCTACTTA | 136 | deletion |
| 263 | TGGAAAAAATCTAAAATCTCCTCAAAAAGTAGAGGTCGACATCATAGATGACAACTTTATCCTGA----GAACAGGAGCGATGGGTCTGTCGGGAATGTGACTTTTTCATTCGATTATCAAAAGTATGTGACTCTACTTA | 136 | deletion |
| 264 | TGGAAAAAATCTAAAATCTCCTCAAAAAGTAGAGGTCGACATCATAGATGACAACTTTATCCTGA----GAACAGGAGCGATGAGTCTTTCGGGAATGTGACTTTTTCATTCGATTATCAAAAGTATGTGACTCTACTTA | 136 | deletion |
| 265 | TGGAAAAAATCTAAAATCTCCTCAAAAAGTAGAGGTCGACATCATAGATGACATCTTTATCCTGAGGTG----AGGAGCGATGAGTCTGTCGGGAATGTGACTTTTTCATTCGATTATCAAAAGTATGTGACTCTACTTA | 136 | deletion |
| 266 | TGGAAAAAATCTAAAATCTCCTCAAAAAGTAGAGGTCGACATCATAGATGACAACTTTATCCTGA----GAACAGGAGCGATGAGTCTGTCGGGAATGTGACTTTTTCATTAGATTATCAAAAGTATGTGACTCTACTTA | 136 | deletion |
| 267 | TGGAAAAAATCTAAAATCTCCTCAAAAAGTAGAGGTCGACACCATAGATGACAACTTTATCCTGA----GAACAGGAGCGATGAGTCTGTCGGGAATGTGACTTTTTCATTCGATTATCAAAAGTATGTGACTCTACTTA | 136 | deletion |
| 268 | TGGAAAAAATCTAAAATCTCCTCAAAAAGTAGAGGTCGACATCATAGATGACAACTTTATCCTGAGGTG----AGGAGCGATGAGTCTGTCGGGAATGTGACTTTTTCATTCGATTATCAAAAGTATGTGACTTTACTTA | 136 | deletion |
| 269 | TGGAAAAAATCTAAAATCTCCTCAAAAAGTAGAGGTCGACATCATAGATGACAACTTTATCCTGAGGTG----AGGAGCGATGAGTCTGTCGGGAATGTGACTTTTTCATTCGATTATCAAAAGTATGTGACTCTACGTA | 136 | deletion |
| 270 | TGGAAAAAATCTAAAATCTCCTCAAAAAGTAGAGGTCGTCATCATAGATGACAACTTTATCCTGAGGTG----AGGAGCGATGAGTCTGTCGGGAATGTGACTTTTTCATTCGATTATCAAAAGTATGTGACTCTACTTA | 136 | deletion |
| 271 | TGGAAAAAATCTAAAATCCCCTCAAAAAGTAGAGGTCGACATCATAGATGACAACTTTATCCTGAGGTG----AGGAGCGATGAGTCTGTCGGGAATGTGACTTTTTCATTCGATTATCAAAAGTATGTGACTCTACTTA | 136 | deletion |
| 272 | TGGAAAAAATCTAAAATCTCCTCAAAAAGTAGAGGTCGACATCATAGATGACAATTTTATCCTGA----GAACAGGAGCGATGAGTCTGTCGGGAATGTGACTTTTTCATTCGATTATCAAAAGTATGTGACTCTACTGA | 136 | deletion |
| 273 | TGGAAAAAATCTAAAATCTCCTCAAAAAGTAGAGGTCGACATCATAGATGACAACTTTATCCTGA----GAACAGGAGCGGTGAGTCTGTCGGGAATGTGACTTTTTCATTCGATTATCAAAAGTATGTGACTCTACTTA | 136 | deletion |
| 274 | TGGAAAAAATCTAAAATCTCCTCAAAAAGTAGAGGCCGACATCATAGATGACAACTTTATCCTGAGGTG----AGGAGCGATGAGTCTGTCGGGAATGTGACTTTTTCATTCGATTATCAAAAGTATGTGACTCTACTTA | 136 | deletion |
| 275 | TGGAAAAAATCTAAAATCTCCTCAAAAAGTAGAGGTCGACATCATAGATGACAACTTTATCCTGA----GAACAGGAGCGATGAGTCTGTCGGGAATGTGACTTTCTCATTCGATTATCAAAAGTATGTGACTCTACTTA | 136 | deletion |
| 276 | TGGAAAAAATCTAAAATCTCCTCAAGAAGTAGAGGTCGACATCATAGATGACAACTTTATCCTGAGGTG----AGGAGCGATGAGTCTGTCGGGAATGTGACTTTTTCATTCGATTATCAAAAGTATGTGACTCTACTTA | 136 | deletion |
| 277 | TGGAAAAAATCTAATATCTCCTCAAAAAGTAGAGGTCGACATCATAGATGACAACTTTATCCTGA----GAACAGGAGCGATGAGTCTGTCGGGAATGTGACTTTTTCATTCGATTATCAAAAGTATGTGACTCTACTTA | 136 | deletion |
| 278 | TGGAAAAAATCTAAAATCTCCTCAAAAAGTAGAGGTCGACATCATAGATGACAACTTTATCCTGA----GAACAGGAGCGATGAGTCTGTCGGGATTGTGACTTTTTCATTCGATTATCAAAAGTATGTGACTCTACTTA | 136 | deletion |
| 279 | TGGAAAAAATCTAAAATCTCCTCAAAAAGTAGAGGTCGACATCATAGATGACAACTTTATCCTGAAGTG----AGGAGCGATGAGTCTGTCGGGAATGTGACTTTTTCATTCGATTATCAAAAGTATGTGACTCTACTTA | 136 | deletion |
| 280 | TGGAAAAAATCTAAAATCTCCTCAAAAAGTAGAGGTCGACATCATAGATGACAACTTTATCCTGA----GAACAGGAGCGATGAGTCTGTCGGGAATGTGACTTTTTCATTCGATTATCAAAAGTATGCGACTCTACTTA | 136 | deletion |
| 281 | TGGAAAAAATCTAAAATCTCCTCAAAAAGTAGAGGTCGACATCATAGATGACAACTTTATCCTGAGGTG----AGGAGCGATGAGTCTGTCGGGAATGTGACTTTTTCATTCGATTATCAAAAGTAGGTGACTCTACTTA | 136 | deletion |
| 282 | TGGAAAAAATCTAAAATCTCCTCAAAAAGTAGAGGTCGACATCATAGAAGACAACTTTATCCTGAGGTG----AGGAGCGATGAGTCTGTCGGGAATGTGACTTTTTCATTCGATTATCAAAAGTATGTGACTCTACTTA | 136 | deletion |
| 283 | TGGAAAAAATCTAAAATCTCCTCAAAAAGTAGAGGTCGACATCATAGATGACAACTTTATCCTGA----GAACAGGAGCGATGAGTCTGTCGGGAATGTGACTTTTTCTTTCGATTATCAAAAGTATGTGACTCTACTTA | 136 | deletion |
| 284 | TGGAAAAAATCTGAAATCTCCTCAAAAAGTAGAGGTCGACATCATAGATGACAACTTTATCCTGA----GAACAGGAGCGATGAGTCTGTCGGGAATGTGACTTTTTCATTCGATTATCAAAAGTATGTGACTCTACTTA | 136 | deletion |
| 285 | TGGAAAAAATCTAAGATCTCCTCAAAAAGTAGAGGTCGACATCATAGATGACAACTTTATCCTGAGGTG----AGGAGCGATGAGTCTGTCGGGAATGTGACTTTTTCATTCGATTATCAAAAGTATGTGACTCTACTTA | 136 | deletion |
| 286 | TGGAAAAAATCTAAAATCTCCTCAAAAAGTAGAGGTCGACATCATAGATGACAACTTTATCCTGA----GAACAGGAGCGATGAGTCTGTCGGGGATGTGACTTTTTCATTCGATTATCAAAAGTATGTGACTCTACTTA | 136 | deletion |
| 287 | TGGAAAAAATCTAAAATCTCCTCAAAAAGTAGAGGTCGACATCATAGATGACAACTTTATCCTGA----GAACCGGAGCGATGAGTCTGTCGGGAATGTGACTTTTTCATTCGATTATCAAAAGTATGTGACTCTACTTA | 136 | deletion |
| 288 | TGGAAAAAATCTAAAATCTCCTCAAAAAGTAGAGGTCGACATCATAGATGACAACTTTATCCTGA----GAACAGGAGAGATGAGTCTGTCGGGAATGTGACTTTTTCATTCGATTATCAAAAGTATGTGACTCTACTTA | 136 | deletion |
| 289 | TGGAAAAAATCTATAATCTCCTCAAAAAGTAGAGGTCGACATCATAGATGACAACTTTATCCTGA----GAACAGGAGCGATGAGTCTGTCGGGAATGTGACTTTTTCATTCGATTATCAAAAGTATGTGACTCTACTTA | 136 | deletion |
| 290 | TGGAAAAAACCTAAAATCTCCTCAAAAAGTAGAGGTCGACATCATAGATGACAACTTTATCCTGAGGTG----AGGAGCGATGAGTCTGTCGGGAATGTGACTTTTTCATTCGATTATCAAAAGTATGTGACTCTACTTA | 136 | deletion |
| 291 | TGGAAAAAATCTAAAATCTCCTCAAAAAGTAGAGGTCGACATCATAGATGACAACTTTATCCTGAGGTG----AGGAGCGATGAGTCTGTCGGGAATGTGATTTTTTCATTCGATTATAAAAAGTATGTGACTCTCCTTA | 136 | deletion |
| 292 | TGGAAAAAATCTAAAATCTCCTCAAAAAGTAGAGGTCGACATCTTAGATGACAACTTTATCCTGAGGTG----AGGAGCGATGAGTCTGTCGGGAATGTGACTTTTTCATTCGATTATCAAAAGTATGTGACTCTACTTA | 136 | deletion |
| 293 | TGGAAAAAATCTAAAATCTCCTCAAAAAGTAGAGGTCGACATCATAGATGACAACTTTATCCTGA----GAGCAGGAGCGATGAGTCTGTCGGGAATGTGACTTTTTCATTCGATTATCAAAAGTATGTGACTCTACTTA | 136 | deletion |
| 294 | TGGAAAAAATCTAAAATCTCCTCAAAAAGTAGAGGTCGACATCATAAATGACAACTTTATCCTGA----GAACAGGAGCGATGAGTCTGTCGGGAATGTGACTTTTTCATTCGATTATCAAAAGTATGTGACTCTACTTA | 136 | deletion |
| 295 | TGGAAAAAATCTAAAATCTCCTTAAAAAGTAGAGGTCGACATCATAGATGACAACTTTATCCTGAGGTG----AGGAGCGATGAGTCTGTCGGGAATGTGACTTTTTCATTCGATTATCAAAAGTATGTGACTCTACTTA | 136 | deletion |
| 296 | TGGAAAAAATCTAAAATCTCCTCAAAAAGTAGAGGTCGACATCATAGATGACAACTTTATCCTGAGGTG----AGGAGCGATGAGTCCGTCGGGAATGTGACTTTTTCATTCGATTATCAAAAGTATGTGACTCTACTTA | 136 | deletion |
| 297 | TGGAAAAAATATAAAATCTCCTCAAAAAGTAGAGGTCGACATCATAGATGACAACTTTATCCTGA----GAACAGGAGCGATGAGTCTGTCGGGAATGTGACTTTTTCATTCGATTATCAAAAGTATGTGACTCTACTTA | 136 | deletion |
| 298 | TGGAAAAAATCTAAAATCTCCTCAAAAAGTAGAGGTCGACATCATAGATGACAACTTTATCCTGA----GAACAGGAGCGATGAGTCTGTCGGGAATGTGACTTTTTCATTCGAATATCAAAAGTATGTGACTCTACTTA | 136 | deletion |
| 299 | TGGAAAAAATCTAAAATCTCCTCAAAAAGTAGAGGTCGACATCATAGATGACAACTTTATCCTGAGGTG----AGGAGCGATGAGTCTGTCGGGAATGTGACTTTTTCATTCGATTATCAAAAGTATGTGACTCTACTAA | 136 | deletion |
| 300 | TGGAAAAAATCTAAAATCTCCTCAAAAAGTAGAGGTCGACATCATGGATGACAACTTTATCCTGAGGTG----AGGAGCGATGAGTCTGTCGGGAATGTGACTTTTTCATTCGATTATCAAAAGTATGTGACTCTACTTA | 136 | deletion |
| 301 | TGGAAAAAATCTAAAATCTCCTCAAAAAGTAGAGGTCGACATCATAGATGACAACTTTATCCTGA----GAACAGGAGCGATGAGTCTGTCGGGAATGTGGCTTTTTCATTCGATTATCAAAAGTATGTGACTCTACTGA | 136 | deletion |
| 302 | TGGAAAAAATCT-AAATCTCCTCAAAAAGTAGAGGTCGACATCATAGATGACAACTTTATCCTGA----GAACAGGAGCGATGAGTCTGTCGGGAATGTGACTTTTTCATTCGATTATCAAAAGTATGTGACTCTACTTA | 135 | deletion |
| 303 | TGGAAAAAATCTAAAATCTCCTCAAAAAGTAGAGGTCGACATCATAGATGACAACTTTATCCTGA----GAACAGGAGCGATGAGTCTGTC-GGAATGTGACTTTTTCATTCGATTATCAAAAGTATGTGACTCTACTTA | 135 | deletion |
| 304 | TGGAAAAAATCTAAAATCTCCTCAAAAAGTAGAGGTCGACATCATAGATGACAACTTTATCCTGA----GAACAGGAGCGATGAGTCTGTCGGGAATGTGACTTTTTCATTCGATTAGCAAAAGTATGTGACTCTACTTA | 136 | deletion |
| 305 | TGGAAAAAATCTAAAATCTCCTCAAAAAGTAGAGGTCGACATCATAGATGACAACTTTATCCTGAGGTG----AGGAGCGATGAGTCTGTCGGGAATGTGACTTTTTCATTCGATTATCAAAAGTATGTTACTCTACTTA | 136 | deletion |
| 306 | TGGAAAAAATCTAAAATCTCCT-AAAAAGTAGAGGTCGACATCATAGATGACAACTTTATCCTGAGGTG----AGGAGCGATGAGTCTGTCGGGAATGTGACTTTTTCATTCGATTATCAAAAGTATGTGACTCTACTTA | 135 | deletion |
| 307 | TGGAAAAAATCTAAAATCTCCTCAAAAAGTAGAGGTCGACATCATAGATGACAACTTTATCCTGA----GAACAGGAGCGACGAGTCTGTCGGGAATGTGACTTTTTCATTCGATTATCAAAAGTATGTGACTCTACTTA | 136 | deletion |
| 308 | TGGAAAAAATCTAAAATCTCCTCAAAAAGTAGAGGTCGACATCATAGATGACAACTTTATCCTGA----GAACAGGAGCGATGAGTCTGTCCGGAATGTGACTTTTTCATTCGATTATCAAAAGTATGTGACTCTACTTA | 136 | deletion |
| 309 | TGGAAAAAATCTAAAATCTCCTCAAAAAGTAGAGGTCGACATCATAGATGACAACTTTATCCTGAGGTG----AGGAGCGATGAGTCTGTCGGGAATGTGACTTTTTCATTCGACTATCAAAAGTATGTGACTCTACTTA | 136 | deletion |
| 310 | TGGAAAAAATCTAAAATCTCCTCAAAAAGTAGAGGTCGACATCATAGATGACAACTTTATCCTG----TGAACAGGAGCGATGAGTCTGTCGGGAATGTGACTTTTTCATTCGATTATCAAAAGTATGTGACTCTACTTA | 136 | deletion |
| 311 | TGGAAAAAATCTAAAATCTCCTCAAAAAGTAGAGGTCGACATCATAGATGACAACTTTATCCTGAGGTG----AGGAGCGACGAGTCTGTCGGGAATGTGACTTTTTCATTCGATTATCAAAAGTATGTGACTCTACTTA | 136 | deletion |
| 312 | TGGAAAAAATCTAAAATCTCCTCAAAAAGTAGAGGTCGACATCATAGATGACTACTTTATCCTGA----GAACAGGAGCGATGAGTCTGTCGGGAATGTGACTTTTTCATTCGATTATCAAAAGTATGTGACTCTACTTA | 136 | deletion |
| 313 | TGGAAAAGATCTAAAATCTCCTCAAAAAGTAGAGGTCGACATCATAGATGACAACTTTATCCTGAGGTG----AGGAGCGATGAGTCTGTCGGGAATGTGACTTTTTCATTCGATTATCAAAAGTATGTGACTCTACTTA | 136 | deletion |
| 314 | TGGAAAAAATCTAAAATCTCCTCAAAAAGTAGAGGTCGACATCATAGATGACAACTTAATCCTGAGGTG----AGGAGCGATGAGTCTGTCGGGAATGTGACTTTTTCATTCGATTATCAAAAGTATGTGACTCTACTTA | 136 | deletion |
| 315 | TGGAAAAAATCTAAAATCTCCTCAAAAAGTAGAGGTCGACATCATAGATGACGACTTTATCCTGAGGTG----AGGAGCGATGAGTCTGTCGGGAATGTGACTTTTTCATTCGATTATCAAAAGTATGTGACTCTACTTA | 136 | deletion |
| 316 | TGGAACAAATCTAAAATCTCCTCAAAAAGTAGAGGTCGACATCATAGATGACAACTTTATCCTGA----GAACAGGAGCGATGAGTCTGTCGGGAATGTGACTTTTTCATTCGATTATCAAAAGTATGTGACTCTACTTA | 136 | deletion |
| 317 | TGGAAAAAATCTAAAATCTCCTCAAAAAGTAGAGGTCGACATCATAGATGACAACTTTATCCTGAGGTG----AGGAGCGGTGAGTCTGTCGGGAATGTGACTTTTTCATTCGATTATCAAAAGTATGTGACTCTACTTA | 136 | deletion |
| 318 | TGGAAAAAATCTAAAATCTCCTCAAAAAGTAGAGGTCGTCATCATAGATGACAACTTTATCCTGA----GAACAGGAGCGATGAGTCTGTCGGGAATGTGACTTTTTCATTCGATTATCAAAAGTATGTGACTCTACTTA | 136 | deletion |
| 319 | TTGAAAAAATCTAAAATCTCCTCAAAAAGTAGAGGTCGACATCATAGATGACAACTTTATCCTGAGGTG----AGGAGCGATGAGTCTGTCGGGAATGTGACTTTTTCATTCGATTATCAAAAGTATGTGACTCTACTTA | 136 | deletion |
| 320 | TGGAAAAAATCTAAAATCTCCTCAAAAAGTAGAGGTCGACATCATAGATGACAACTTTATCCTGAGGTG----AGGAGCGATGAGTCTGTCTGGAATGTGACTTTTTCATTCGATTATCAAAAGTATGTGACTCTACTTA | 136 | deletion |
| 321 | TGGAAAAAATCTAAAATCTCCTCAAAAAGTAGAGGTCGACATCATAGATGACAACTTTATCCTGA----GAACAGGAGCGATGAGTCTGTCGGGAATGTGACTTTTTCATTCGATTATCAAAAGTATGTGACTCAACTTA | 136 | deletion |
| 322 | TGGAAAAAATCTAAAATCTCCTCAAAAAGTAGAGGTCGACATCATAGATGACAACTTTATCCTGAGGTG----AGGAGCGATGAGTCTGTCGGGAATGTGACTTTTTCATTCGATTATAAAAAGTATGTGACTCTACTGA | 136 | deletion |
| 323 | TGGAAAAAATCTAAAATCTCCTCAAAAAGTAGAGGTCGACATCATAGATGACAACTTTATCCTGA----GAACAGGAGCGATGAGTCTGTCGGGAATGTGACTTTTCCATTCGATTATAAAAAGTATGTGACTCTACTTA | 136 | deletion |
| 324 | TGGAAAAAATCTAAAATCTCCTCAAAAAGTAGAGGTCGACATCATAGATGACAACTTTATCCTGA----GAACAGGAGCGATGAGTCTGTCGGGAATGTGACTTTTTCATTCGATTATCAAAAGTATGTGACTCTAGTTA | 136 | deletion |
| 325 | TGGAAAAAATCTAAAATCTCCTCAAAAAGTAGAGGTCGACGTCATAGATGACAACTTTATCCTGAGGTG----AGGAGCGATGAGTCTGTCGGGAATGTGACTTTTTCATTCGATTATCAAAAGTATGTGACTCTACTTA | 136 | deletion |
| 326 | TGGAAAAAATCTAAAATCTCCTCAAAAAGTAGAGGTCGACATCATAGATGACAACTTTATCCTGAGGTG----AGGAGCGATGAGTCTGTCGGGAATGTGAATTTTTCATTCGATTATCAAAAGTATGTGACTCTACTGA | 136 | deletion |
| 327 | TGGAAAAAATATAAAATCTCCTCAAAAAGTAGAGGTCGACATCATAGATGACAACTTTATCCTGAGGTG----AGGAGCGATGAGTCTGTCGGGAATGTGACTTTTTCATTCGATTATCAAAAGTATGTGACTCTACTTA | 136 | deletion |
| 328 | TGGAAAAAATCTAAAATCTCCTCAAAAAGTAGAGGTCGACATCATAGATGACAACTTTATCCTGA----GAACAGGAGCGATGAGTCTGTCGGGAGTGTGACTTTTTCATTCGATTATCAAAAGTATGTGACTCTACTTA | 136 | deletion |
| 329 | TGGAAAAAATCTAAAATCTCCTCAAAAAGTAGAGGTCGACATCATAGATGACAACTTTATCCTGA----GAACAGGAGCGATGAGTATGTCGGGAATGTGACTTTTTCATTCGATTATCAAAAGTATGTGACTCTACTTA | 136 | deletion |
| 330 | TGGAAAAAATCTAAAATCTCCTCAAAAAGTAGAGGTCGACGTCATAGATGACAACTTTATCCTGA----GAACAGGAGCGATGAGTCTGTCGGGAATGTGACTTTTTCATTCGATTATCAAAAGTATGTGACTCTACTTA | 136 | deletion |
| 331 | TGGAAAAAATCTAAAACCTCCTCAAAAAGTAGAGGTCGACATCATAGATGACAACTTTATCCTGAGGTG----AGGAGCGATGAGTCTGTCGGGAATGTGACTTTTTCATTCGATTATCAAAAGTATGTGACTCTACTTA | 136 | deletion |
| 332 | TGGAAAAAATCTAAAATCTCCTCAAATAGTAGAGGTCGACATCATAGATGACAACTTTATCCTGA----GAACAGGAGCGATGAGTCTGTCGGGAATGTGACTTTTTCATTCGATTATCAAAAGTATGTGACTCTACTTA | 136 | deletion |
| 333 | TGGAAAAAATCTAAAATCTCCTCAAAAAGTAGAGGTCGACATCATAGATGACAACTTTATCCTGAGGTG----AGGAGCGATGAGTCTGTCGGGAATTTGACTTTTTCATTCGATTATCAAAAGTATGTGACTCTACTTA | 136 | deletion |
| 334 | TGGGAAAAATCTAAAATCTCCTCAAAAAGTAGAGGTCGACATCATAGATGACAACTTTATCCTGA----GAACAGGAGCGATGAGTCTGTCGGGAATGTGACTTTTTCATTCGATTATCAAAAGTATGTGACTCTACTTA | 136 | deletion |
| 335 | TGGAAAAAATCTAAAATCTCCTCAAAAAGTAGAGGTCGACATCATAGATGACAACTTTATCCTGAGGTG----ATGAGCGATGAGTCTGTCGGGAATGTGACTTTTTCATTCGATTATCAAAAGTATGTGACTCTACTTA | 136 | deletion |
| 336 | TGGAAAAAATCTAAAATCTCCTCAAAAAGTAGAGGTCGACATCATAGATGACAACTTTATCCTGAGGTG----AGGAGCGATGAGTCTGTCGGGAATGTGCCTTTTTCATTCGATTATCAAAAGTATGTGACTCTACTTA | 136 | deletion |
| 337 | TGGAAAAAATCTAAAATCTCCTCAAAAAGTAGAGGTCGACACCATAGATGACAACTTTATCCTGAGGTG----AGGAGCGATGAGTCTGTCGGGAATGTGACTTTTTCATTCGATTATCAAAAGTATGTGACTCTACTTA | 136 | deletion |
| 338 | TGGAAAAAATCTAAAATCTCCTCAAAAAGTAGAGGACGACATCATAGATGACAACTTTATCCTGAGGTG----AGGAGCGATGAGTCTGTCGGGAATGTGACTTTTTCATTCGATTATCAAAAGTATGTGACTCTACTTA | 136 | deletion |
| 339 | TGGAAAAAATCT-AAATCTCCTCAAAAAGTAGAGGTCGACATCATAGATGACAACTTTATCCTGAGGTG----AGGAGCGATGAGTCTGTCGGGAATGTGACTTTTTCATTCGATTATCAAAAGTATGTGACTCTACTTA | 135 | deletion |
| 340 | TGGAAAAAATCTAAAATATCCTCAAAAAGTAGAGGTCGACATCATAGATGACAACTTTATCCTGAGGTG----AGGAGCGATGAGTCTGTCGGGAATGTGACTTTTTCATTCGATTATCAAAAGTATGTGACTCTACTTA | 136 | deletion |

| | | | |
|---|---|---|---|
| 341 | TGGAAAAAATCTAAAATCTCCTCAAAAAGTAGAGGTCGACATCATAGATGACAACTTTATCCTGA----GAACAGGAGCGATGAGTCTGTCGGGAATGTGACTTTTTCATTCGATTATCAAAAGTATGTGACTCTAATTA | 136 | deletion |
| 342 | TGGAAAAAATCTAAAATCTCCTCAAAAAGTAGAGGTCGACATCATAGATGACAACTTTATCCTGAGGTG----AGGAGCGATGAGTCTGTCGGGAATGTGACTTTCTCATTCGATTATCAAAAGTATGTGACTCTACTTA | 136 | deletion |
| 343 | TGGAAAAAATCTAAAATCTCCTCAAAAAGTAGAGGTCGACATCATAGATGACAACTTTATCCTGA----GAACAGGAGCGATGAGTCTGTCGGGAATGTGACTTTTTCATTCGATTATCAGAAGTATGTGACTCTACTTA | 136 | deletion |
| 344 | TGGAAAAAATCTAAAATCTCCTCAAAAAGTAGAGGTCGACATCATAGATGACAACTTTATCCTGA----GAACAGGAGCGATGAGTCTGTCGGGAATGTGACTTTTTCATTCGATTATCAAAAGTAGGTGACTCTACTTA | 136 | deletion |
| 345 | TGGAAAAAATCTAAAATCTCCTCAAAAAGTAGAGGTCGACATCATAGATGACAACTTTATCCTGAGGTG----AGGAGCGATGAGTCTGTCGGGAATGTGACTTTTTCATTCGATCATCAAAAGTATGTGACTCTACTTA | 136 | deletion |
| 346 | TGGAAAAAATCTAAAATCTCCTCAAAAAGTAGAGGTCGACATCATAGATTACAACTTTATCCTGAGGTG----AGGAGCGATGAGTCTGTCGGGAATGTGACTTTTTCATTCGATTATCAAAAGTATGTGACTCTACTTA | 136 | deletion |
| 347 | TGGAAAAAATCTAAAATCTCCTCAAAAAGTAGAGGTCGACATCATAGATGACAACTTTATCCTGA----GAAGAGGAGCGATGAGTCTGTCGGGAATGTGACTTTTTCATTCGATTATCAAAAGTATGTGACTCTACTTA | 136 | deletion |
| 348 | TGGAAAAAATCTAAAATCTCCTCAAAAAGTAGAGGTCGACATCATAGATGACAACTTTATCCTGA----GAACAGGAGCGATGAGTCTGTCGGGAACGTGACTTTTTCATTCGATTATCAAAAGTATGTGACTCTACTTA | 136 | deletion |
| 349 | TGGAAAAAATCTAAAATCTCCTCAAAAAGTAGAGGTCGACATCATAGATGACAACTTTATCCTGAGGTG----AGGAGCGATGAGGCTGTCGGGAATGTGACTTTTTCATTCGATTATCAAAAGTATGTGACTCTACTTA | 136 | deletion |
| 350 | TGGAAAAAATCTAAAATCTCCTCAAAAAGTAGAGGTCGACATCATAGATGACAACTTTATCCTGA----GAACAGGAGCGATGAGTCTGTCGGGAATGTGACTTTTTCATTCGA-TATCAAAAGTATGTGACTCTACTTA | 135 | deletion |
| 351 | TGGAAAAAATCTAAAATCTCCTCAAAAAGTAGAGGTCGACATCATAGATGACAACTTTATCCTGAGGTG----AGGAGCGATGAGTCTGTCGGGAGTGTGACTTTTTCATTCGATTATCAAAAGTATGTGACTCTACTTA | 136 | deletion |
| 352 | TGGAAAAAATCTAAAATCTCCTCAAAAAGTAGAGGTGGACATCATAGATGACAACTTTATCCTGAGGTG----AGGAGCGATGAGTCTGTCGGGAATGTGACTTTTTCATTCGATTATCAAAAGTATGTGACTCTACTTA | 136 | deletion |
| 353 | TGGAAAAAATCTAAAATCTCCTCAAAAAGTAGAGGTCGACATCATAGATGACAACTTTATCCTGA----GAACAGGAACGATGAGTCTGTCGGGAATGTGACTTTTTCATTCGATTATCAAAAGTATGTGACTCTACTGA | 136 | deletion |
| 354 | TGGAAAAAGTCTAAAATCTCCTCAAAAAGTAGAGGTCGACATCATAGATGACAACTTTATCCTGAGGTG----AGGAGCGATGAGTCTGTCGGGAATGTGACTTTTTCATTCGATTATCAAAAGTATGTGACTCTACTTA | 136 | deletion |
| 355 | TGGATAAAATCTAAAATCTCCTCAAAAAGTAGAGGTCGACATCATAGATGACAACTTTATCCTGA----GAACAGGAGCGATGAGTCTGTCGGGAATGTGACTTTTTCATTCGATTATCAAAAGTATGTGACTCTACTTA | 136 | deletion |
| 356 | TGGAAAAAATCTAAAATCTCCTCAAAAAGTAGAGGTCGACATCATAGATGACATCTTTATCCTGA----GAACAGGAGCGATGAGTCTGTCGGGAATGTGACTTTTTCATTCGATTATCAAAAGTATGTGACTCTACTTA | 136 | deletion |
| 357 | TGGAAAAAATCTAAAATCTCCTCAAAAAGTAGAGGTCGACATCATAGATGACAACTTTATCCTGAGGTG----AGGAGCGATGAGTCTGTCGGGAATGTGACTTTTTCATCCGATTATCAAAAGTATGTGACTCTACTTA | 136 | deletion |
| 358 | TGGAAAAAATCTAAAATCTCCTCAAAAAGTAGAGGTCGACATCATAGATGACAACTTTATCCTGA----GAACAGGAGCGATGAGTCTGTCGGGAATGTGACTTTTTCATTGGATTATCAAAAGTATGTGACTCTACTTA | 136 | deletion |
| 359 | TGGAAAAAATCTAAAATCTCCTCAAAAAGTAGAGGTCCACATCATAGATGACAACTTTATCCTGA----GAACAGGAGCGATGAGTCTGTCGGGAATGTGACTTTTTCATTCGATTATCAAAAGTATGTGACTCTACTTA | 136 | deletion |
| 360 | TGGAAAAAATCTAAAATCTCCTCAAAAAGTAGAGGTCGACATCAAAGATGACAACTTTATCCTGA----GAACAGGAGCGATGAGTCTGTCGGGAATGTGACTTTTTCATTCGATTATCAAAAGTATGTGACTCTACTTA | 136 | deletion |
| 361 | TGGAAAAAATCTAAAATCTCCTCAAAAAGTAGAGGTCGACATCATAGATGACAACTTTATCCTGA----GAACAGGAGCGATGAGTCTGTCGGGAATGTGACTTTTTAATTCGATTATAAAAAGTATGTGACTCTACTTA | 136 | deletion |
| 362 | TGGAAAAAATCTAAAATCTCCTCAGAAAGTAGAGGTCGACATCATAGATGACAACTTTATCCTGAGGTG----AGGAGCGATGAGTCTGTCGGGAATGTGACTTTTTCATTCGATTATCAAAAGTATGTGACTCTACTTA | 136 | deletion |
| 363 | TGGAAAAAATCTAAAATCTCCTCAAAAAGTAGAGGTCGACATCACAGATGACAACTTTATCCTGA----GAACAGGAGCGATGAGTCTGTCGGGAATGTGACTTTTTCATTCGATTATCAAAAGTATGTGACTCTACTTA | 136 | deletion |
| 364 | TGGAAAATATCTAAAATCTCCTCAAAAAGTAGAGGTCGACATCATAGATGACAACTTTATCCTGA----GAACAGGAGCGATGAGTCTGTCGGGAATGTGACTTTTTCATTCGATTATCAAAAGTATGTGACTCTACTTA | 136 | deletion |
| 365 | TGGAAAAAATCTAAAATCTCCTCAAAAAGTGGAGGTCGACATCATAGATGACAACTTTATCCTGA----GAACAGGAGCGATGAGTCTGTCGGGAATGTGACTTTTTCATTCGATTATCAAAAGTATGTGACTCTACTGA | 136 | deletion |
| 366 | TGGAAAAAATCTAAAATCTCCTCAAAAAGTAGAGGTCGACATCATAGATGACAACTTTATCCTGGGGTG----AGGAGCGATGAGTCTGTCGGGAATGTGACTTTTTCATTCGATTATCAAAAGTATGTGACTCTACTTA | 136 | deletion |
| 367 | TGGAAAAAATCTAAAATCTCCTCAAAAAGTAGAGGTCGACATCATAGATGACAACTTTATCCTGA----GAACAGGAGGGATGAGTCTGTCGGGAATGTGACTTTTTCATTCGATTATCAAAAGTATGTGACTCTACTTA | 136 | deletion |
| 368 | TGGAAAAAATCTAAAATCTCCTCAAAAAGTAGAGGTCGACATCATAGATGACAACTTTATCCTGAGGTG----AGGAGCGATGAGTCTGTCGGGAATGTGACTTTTTCATTCGATTATCAAAAGCATGTGACTCTACTTA | 136 | deletion |
| 369 | GGGAAAAAATCTAAAATCTCCTCAAAAAGTAGAGGTCGACATCATAGATGACAACTTTATCCTGAGGTG----AGGAGCGATGAGTCTGTCGGGAATGTGACTTTTTCATTCGATTATCAAAAGTATGTGACTCTACTGA | 136 | deletion |
| 370 | TGGAAAAAATCTAAAATCTCCTCAAAAAGTAGAGGTCGACATCATAGATGACAACTTTATCCTGAGGTG----AGGAGCGATGAGTCTGTCTGGAATGTGACTTTTTCATTCGATTATCAAAAGTATGTGACTCTACTGA | 136 | deletion |
| 371 | TGGAAAAAATCTAAAATCTCCTCAAAAAGTAGAGGTCGACATCATAGATGACAACTTTATCCTGA----GAACAGGAGCAATGAGTCTGTCGGGAATGTGACTTTTTCATTCGATTATCAAAAGTATGTGACTCTACTGA | 136 | deletion |
| 372 | TGGAAAAAATCTAAAATCTCCTCAAAAAGTAGAGGTCGACATCATAGATGATAACTTTATCCTGAGGTG----AGGAGCGATGAGTCTGTCGGGAATGTGACTTTTTCATTCGATTATCAAAAGTATGTGACTCTACTGA | 136 | deletion |
| 373 | TGGAAAAAATCTAAAATCTCCTCAAAAAGTAGAGGTCGACATCATAGATGACAACTCTATCCTGA----GAACAGGAGCGATGAGTCTGTCGGGAATGTGACTTTTTCATTCGATTATCAAAAGTATGTGACTCTACTTA | 136 | deletion |
| 374 | TGGAAAAAATCTAAAATCTCCTCGAAAAGTAGAGGTCGACATCATAGATGACAACTTTATCCTGA----GAACAGGAGCGATGAGTCTGTCGGGAATGTGACTTTTTCATTCGATTATCAAAAGTATGTGACTCTACTTA | 136 | deletion |
| 375 | TGGAAAAAATCTAAAATCTCCTCAAAAAGTAGAGGTCGACATCATAGATGACAACTTTATCCTGAGGTG----AGGAGCGATGAGTCTGTCGGGAATGTGATTTTTTCATTCGATTATCAAAAGTATGTGACTCTACTGA | 136 | deletion |
| 376 | TGGAAAAACTCTAAAATCTCCTCAAAAAGTAGAGGTCGACATCATAGATGACAACTTTATCCTGAGGTG----AGGAGCGATGAGTCTGTCGGGAATGTGACTTTTTCATTCGATTATCAAAAGTATGTGACTCTACTTA | 136 | deletion |
| 377 | TGGAAAAAATCTAAAATCTCCTCAAAAAGTAGAGGTCGACATCATAGATGACAACTTTATCCTGAGGTG----AGGAGCGATGAGCCTGTCGGGAATGTGACTTTTTCATTCGATTATCAAAAGTATGTGACTCTACTGA | 136 | deletion |
| 378 | TGGAAAAAATCTAAAATCTCCTCAAAAAGTAGAGGTCGACATCATAGATGACAACTTTATCCTGA----GAACAGGAGCGATGAGTCTGTCGGGAATGTGACTTTTTCATTCGACTATCAAAAGTATGTGACTCTACTTA | 136 | deletion |
| 379 | TGGAAAAAATCTAAAATCTCCTCAAAAAGTAGAGGTCAACATCATAGATGACAACTTTATCCTGA----GAACAGGAGCGATGAGTCTGTCGGGAATGTGACTTTTTCATTCGATTATCAAAAGTATGTGACTCTACTGA | 136 | deletion |
| 380 | TGGAAAAAATCTAAAATCTCCTCAAAAAGTAGAGGTCGACATCATAGATGACAACTTTATCCTGAGGTG----AGGAGCGAAGAGTCTGTCGGGAATGTGACTTTTTCATTCGATTATCAAAAGTATGTGACTCTACTTA | 136 | deletion |
| 381 | TGGAAAAAATCTAAAATCTCCTCAAAAAGTAGAGGTCGACATCATAGATGACAACTTTATCCTGA----GAACAGGAGCGATGAGTCTGTCGGGAATGTGACTTTTTCATTCGATTATCAAAAGTATGTGCCTCTACTTA | 136 | deletion |
| 382 | TGGAAAAAATCTAAAATCTCCTCAAAAAGTAGAGGTCGACATCATAGATGACAACTTTATCCTGA----GAACAGGAGCGATGAGTCTGTCGGGAATGTTACTTTTTCATTCGATTATCAAAAGTATGTGACTCTACTTA | 136 | deletion |
| 383 | TGGAAAAAATCTAAAATCTCCTCAAAAAGTAGAGGTCGACATCATATATGACAACTTTATCCTGAGGTG----AGGAGCGATGAGTCTGTCGGGAATGTGACTTTTTCATTCGATTATCAAAAGTATGTGACTCTACTTA | 136 | deletion |
| 384 | TGGAAAAAATCTAAAATCTCCTCAAAAAGTAGAGGTCGACATCATAGATGACAACTTTATCCAGA----GAACAGGAGCGATGAGTCTGTCGGGAATGTGACTTTTTCATTCGATTATCAAAAGTATGTGACTCTACTTA | 136 | deletion |
| 385 | TGGAAAAAATCTAAAATCTCCTCAAAAAGTAGAGGTCGACATCATAGATGACAACTTTATCCTGAGGTG----AGGAGCGATAAGTCTGTCGGGAATGTGACTTTTTCATTCGATTATCAAAAGTATGTGACTCTACTGA | 136 | deletion |
| 386 | TGGAAAAAATCTAAAATCTCCTCAAAATGTAGAGGTCGACATCATAGATGACAACTTTATCCTGAGGTG----AGGAGCGATGAGTCTGTCGGGAATGTGACTTTTTCATTCGATTATCAAAAGTATGTGACTCTACTTA | 136 | deletion |
| 387 | TGGAAAAAAACTAAAATCTCCTCAAAAAGTAGAGGTCGACATCATAGATGACAACTTTATCCTGA----GAACAGGAGCGATGAGTCTGTCGGGAATGTGACTTTTTCATTCGATTATCAAAAGTATGTGACTCTACTTA | 136 | deletion |
| 388 | TGGAAAAAATCTAAAATCTCCTCAAAAAGTAGAGGTCGACATCATAGATGACAACTTTATCCTGAGGTG----AGGAGCGATGAGTCTGTCGGGAATGTGACTTTTTCATTCGATT-TCAAAAGTATGTGACTCTACTTA | 135 | deletion |
| 389 | TGGAAAAAATCTAAAATCTCCTCAAAAAGTAGAGGTCGACATCATAGATAACAACTTTATCCTGA----GAACAGGAGCGATGAGTCTGTCGGGAATGTGACTTTTTCATTCGATTATCAAAAGTATGTGACTCTACTGA | 136 | deletion |
| 390 | TGGAAAAAATCTAAAATCTCCTCAAAAAGTAGAGGTCGACATCATAGATGACAACTTTATCCTGAGGTG----AGGAGCGATGAGTCTGTCGGGAATGTGACTTTTTCATTCGATTATCAAAAGTATGTGACTCTACTCA | 136 | deletion |
| 391 | TGGAAAAAATCTAAAATCTCCTCAAAAAGTAGAGGTCGACATCATAGTTGACAACTTTATCCTGA----GAACAGGAGCGATGAGTCTGTCGGGAATGTGACTTTTTCATTCGATTATCAAAAGTATGTGACTCTACTTA | 136 | deletion |
| 392 | TGGAAAAAATCTAAAATCTCCTCAAAAAGTAGAGGTCGACATCATAGATGACAACTTTATCCTGAGGTG----AGGAGCGATGAGTCTGTCGGGAATGTGACTTTTTCATTCGATTATCAAAAGTACGTGACTCTACTTA | 136 | deletion |
| 393 | TGGAAAAAATCTAAAATCTCCTCAAAAAGTAGAGGTCGACATCATAGATGACAACTTTATCCTGAGGTG----AGGAGCGATGAGTCTGTCGGGAATGTGACTTATTCATTCGATTATCAAAAGTATGTGACTCTACTTA | 136 | deletion |
| 394 | TGGAAAAAATCTAAAATCTCCTCAAAAAGTAGAGGTCGACATCATAGATGACAACTTTATCCTGAGGTG----AGGAGCGATGAGTCTGTCGGG-ATGTGACTTTTTCATTCGATTATCAAAAGTATGTGACTCTACTTA | 135 | deletion |
| 395 | TGGCAAAAATCTAAAATCTCCTCAAAAAGTAGAGGTCGACATCATAGATGACAACTTTATCCTGA----GAACAGGAGCGATGAGTCTGTCGGGAATGTGACTTTTTCATTCGATTATCAAAAGTATGTGACTCTACTTA | 136 | deletion |
| 396 | TGGAAAAAATCTAAAATCTCCTCAAAAAGTAGAGGTCGACATCATAGATGACAGCTTTATCCTGA----GAACAGGAGCGATGAGTCTGTCGGGAATGTGACTTTTTCATTCGATTATCAAAAGTATGTGACTCTACTTA | 136 | deletion |
| 397 | TGGAAAAAATCTAAAATCTCCTCAAAAAGTAGAGGTCGACATCATAGATGACAACTATATCCTGA----GAACAGGAGCGATGAGTCTGTCGGGAATGTGACTTTTTCATTCGATTATCAAAAGTATGTGACTCTACTTA | 136 | deletion |
| 398 | TGGAAAAAATCTAAAACCTCCTCAAAAAGTAGAGGTCGACATCATAGATGACAACTTTATCCTGA----GAACAGGAGCGATGAGTCTGTCGGGAATGTGACTTTTTCATTCGATTATCAAAAGTATGTGACTCTACTTA | 136 | deletion |
| 399 | TGGAAAAAATCTAAAATCTCCTCAAAAAGTAGAGGTCGACATCATAGATGACAACTTTATCCTGA----GAACAGGAGCGATGAGTCTGTCGGGAATGTGACTTTTTCATCCGATTATCAAAAGTATGTGACTCTACTTA | 136 | deletion |
| 400 | TGGAAAAAATCTAAAATCCCCTCAAAAAGTAGAGGTCGACATCATAGATGACAACTTTATCCTGAGGTG----AGGAGCGATGAGTCTGTCGGGAATGTGACTTTTTCATTCGATTATCAAAAGTATGTGACTCTACTGA | 136 | deletion |
| 401 | TGGAAAAAATCTAAAATCTCCTCAAAAAGTAGAGGTCGACATCATAGATGACAACTTCATCCTGAGGTG----AGGAGCGATGAGTCTGTCGGGAATGTGACTTTTTCATTCGATTATCAAAAGTATGTGACTCTACTTA | 136 | deletion |
| 402 | TGGAAAAAATCTAAAATCTCCTCAAAAAGTAGAGGTCGACATCATAGATGACAACTTTATCCTGAGGTG----AGGAGCGATGAGTCTGTCGGGAATGTGACTTTTTCATTCGATTATCAAAAGTATGTGACTCGACTTA | 136 | deletion |
| 403 | TGGAAAAAATCTAAAATCTCCTCAAAAAGTAGAGGTCGACATCATAGAGGACAACTTTATCCTGAGGTG----AGGAGCGATGAGTCTGTCGGGAATGTGACTTTTTCATTCGATTATCAAAAGTATGTGACTCTACTTA | 136 | deletion |
| 404 | TGGAAAAAATCTAAAATCTCCTCAAAAAGTATAGGTCGACATCATAGATGACAACTTTATCCTGAGGTG----AGGAGCGATGAGTCTGTCGGGAATGTGACTTTTTCATTCGATTATCAAAAGTATGTGACTCTACTTA | 136 | deletion |
| 405 | TGGAAAAAATCTAATATCTCCTCAAAAAGTAGAGGTCGACATCATAGATGACAACTTTATCCTGAGGTG----AGGAGCGATGAGTCTGTCGGGAATGTGACTTTTTCATTCGATTATCAAAAGTATGTGACTCTACTTA | 136 | deletion |
| 406 | TGGAAAAAATCTAAAATCTCCTCAAAAAGTAGAGGTCGACATCATAGATGACAACTTTATCCTGA----GAACAGGAGCGATGAGTCTGTCGGGAATGTGACTTTTTCATTCGATTATCAATAGTATGTGACTCTACTTA | 136 | deletion |
| 407 | TGGAAAAAATCTAAAATCTCCTCAAAAAGTAGAGGTCGACATCATAGATGACAACTTTATCCTGA----GAACAGGAGCGATGAGTCTGTCGGGAATGTGACTTTTTCATTCGATTATCAAAAGTAAGTGACTCTACTTA | 136 | deletion |
| 408 | TGGAGAAAATCTAAAATCTCCTCAAAAAGTAGAGGTCGACATCATAGATGACAACTTTATCCTGA----GAACAGGAGCGATGAGTCTGTCGGGAATGTGACTTTTTCATTCGATTATCAAAAGTATGTGACTCTACTTA | 136 | deletion |
| 409 | TGGAAAAAATCTAAAATCTCCTCAAAAAGTAGAGGTCGACATCATAGATGACAACTTTATCCTGAGGTG----AGGAGCGATGAGTCTGTCGGGAATGTGACTTTTTCATACGATTATCAAAAGTATGTGACTCTACTGA | 136 | deletion |
| 410 | TGGAAAAAATCTAAAATCTCCTCAAAAAGTAGAGGTCGACATCATATATGACAACTTTATCCTGA----GAACAGGAGCGATGAGTCTGTCGGGAATGTGACTTTTTCATTCGATTATCAAAAGTATGTGACTCTACTTA | 136 | deletion |
| 411 | TGGAAAAAATCTAAAATCTCCTCAAAAAGTAGAGGTCGACATCATAGATGACAACTTTATCCTGAGGTG----AGGAGCGATGAGTCTGTCGGGAATGTGACTCTTTCATTCGATTATCAAAAGTATGTGACTCTACTTA | 136 | deletion |
| 412 | TGGAAGAAATCTAAAATCTCCTCAAAAAGTAGAGGTCGACATCATAGATGACAACTTTATCCTGAGGTG----AGGAGCGATGAGTCTGTCGGGAATGTGACTTTTTCATTCGATTATCAAAAGTATGTGACTCTACTTA | 136 | deletion |
| 413 | TGGAAAAAATCTAAAATCTCCTCAAAAAGTAGAGGTCGACATCATAGATGACAACTTTATCCTGAGGTG----AGGAGCGATGAGTCTGTCGGGAATGTGACTTTTTCATTCGATTATCAATAGTATGTGACTCTACTTA | 136 | deletion |
| 414 | TGGTAAAAATCTAAAATCTCCTCAAAAAGTAGAGGTCGACATCATAGATGACAACTTTATCCTGA----GAACAGGAGCGATGAGTCTGTCGGGAATGTGACTTTTTCATTCGATTATCAAAAGTATGTGACTCTACTGA | 136 | deletion |
| 415 | TGGAAAAAATCTAAAATCTCCTCAAAAAGTAGAGGTCGACATCATAGATGACAACTTTATCCTGAGGTG----AGGAGCGAGGAGTCTGTCGGGAATGTGACTTTTTCATTCGATTATCAAAAGTATGTGACTCTACTTA | 136 | deletion |
| 416 | TGGAAAAAATCTAAAATCTCCTCAAAAAGTTGAGGTCGACATCATAGATGACAACTTTATCCTGA----GAACAGGAGCGATGAGTCTGTCGGGAATGTGACTTTTTCATTCGATTATCAAAAGTATGTGACTCTACTTA | 136 | deletion |
| 417 | TGGAAAAAATCTAAAATCTCCTCAAAAAGTAGAGGTCGACATCATAGATGACAACTTTATCCTGAGGTG----AGGAGCGATGAGTCTGTCGGAAATGTGACTTTTTCATTCGATTATAAAAAGTATGTGACTCTACTTA | 136 | deletion |
| 418 | TGGAAAAAATCTAAAATCTCCTCAAAAAGTAGAGGTCGACATCATAGATGACAACTTTATCCTGA----GAACAGGAGCGATGAGTCTGTCGGGAATGTGACTTTATCATTCGATTATCAAAAGTATGTGACTCTACTTA | 136 | deletion |
| 419 | CGGAAAAAATCTAAAATCTCCTCAAAAAGTAGAGGTCGACATCATAGATGACAACTTTATCCTGAGGTG----AGGAGCGATGAGTCTGTCGGGAATGTGACTTTTTCATTCGATTATCAAAAGTATGTGACTCTACTTG | 136 | deletion |
| 420 | TGGAAAAAATCTAAATTCTCCTCAAAAAGTAGAGGTCGACATCATAGATGACAACTTTATCCTGAGGTG----AGGAGCGATGAGTCTGTCGGGAATGTGACTTTTTCATTCGATTATCAAAAGTATGTGACTCTACTTA | 136 | deletion |
| 421 | TGGAAAAAATCTAAAATCTCCTCAAAAAGTAGAGGTCGACATCATAGATGACAACTTTATCCTGA----GAACAGGAGCGATGAGTCTGTCGGGAATGTGACTTTTTCATTCGATTATCAAAAGTATGTGACTGTACTTA | 136 | deletion |
| 422 | TGGAAAAAATCTAAAATCTCCTCAAAAAGTAGTGGTCGACATCATAGATGACAACTTTATCCTGA----GAACAGGAGCGATGAGTCTGTCGGGAATGTGACTTTTTCATTCGATTATCAAAAGTATGTGACTCTACTTA | 136 | deletion |
| 423 | TGGAAAAAATCTAAAATCTCCTCAAAAAGTAGAGGTCGACATCATAGATGACAACTTTATCCTGAGGTG----AGGAGCTATGAGTCTGTCGGGAATGTGACTTTTTCATTCGATTATCAAAAGTATGTGACTCTACTTA | 136 | deletion |
| 424 | TGGAAAAAATCTAAAATCTCCTCAAAAAGTAGAGGTAGACATCATAGATGACAACTTTATCCTGAGGTG----AGGAGCGATGAGTCTGTCGGGAATGTGACTTTTTCATTCGATTATCAAAAGTATGTGACTCTACTTA | 136 | deletion |
| 425 | TGGAAAAAATCTAAAATCTCCTCAAAAAGTAGAGGTCGACATCATAGATGACAACTTTATCCTGA----GAACAGGAGCGATTAGTCTGTCGTGAATGTGACTTTTTCATTCGATTATCAAAAGTATGTGACTCTACTTA | 136 | deletion |
| 426 | TGGAAAAAATCTAAAATCTCCTCAAAAAGTAGAGGTCGACATCAAAGATGACAACTTTATCCTGAGGTG----AGGAGCGATGAGTCTGTCGGGAATGTGACTTTTTCATTCGATTATCAAAAGTATGTGACTCTACTTA | 136 | deletion |

| | | | |
|---|---|---|---|
| 427 | TGGAAAAAATCTAAAATCTCCTCAAAAAGTAGAGGTCGACATCATAGATGACAACTTTATCCTGA----GAACAAGAGCGATGAGTCTGTCGGGAATGTGACTTTTTCATTCGATTATCAAAAGTATGTGACTCTACTGA | 136 | deletion |
| 428 | TGGAAAAAATCTAAAATCTCATCAAAAAGTAGAGGTCGACATCATAGATGACAACTTTATCCTGA----GAACAGGAGCGATGAGTCTGTCGGGAATGTGACTTTTTCATTCGATTATCAAAAGTATGTGACTCTACTTA | 136 | deletion |
| 429 | AGGAAAAAATCTAAAATCTCCTCAAAAAGTAGAGGTCGACATCATAGATGACAACTTTATCCTGAGGTG----AGGAGCGATGAGTCTGTCGGGAATGTGACTTTTTCATTCGATTATCAAAAGTATGTGACTCTACTGA | 136 | deletion |
| 430 | TGGAAAAAATCTAAAATCTCCTCAAAAAGTAGAGGTCGACATCATAGATGACAACTTTATCCTGAGGTG----AGGAGCGATGAGTCTGTCGGGAATGTGACTTTTTAATTCGATTATCAAAAGTATGTGACTCTACTTA | 136 | deletion |
| 431 | TGGAAAAAATCTAAAATCTCCTCAAAAAGTAGAGGTCGACATCATAGATGACAACTTTAACCTGAGGTG----AGGAGCGATGAGTCTGTCGGGAATGTGACTTTTTCATTCGATTATCAAAAGTATGTGACTCTACTTA | 136 | deletion |
| 432 | TGGAAAAAATCTAAAATCTCCTCAAAAAGTAGAGGTCGACATCATAGATGACAACTTTATCCTGA----GAACAGGAGCGATGAGTCTGTCGGGAATGTGACTTTTTCATTCGATTATCAAAAGTATGTGACTCTACTAA | 136 | deletion |
| 433 | TGGAAAAAATCTAAAATCTCCTCAAAAAGTAGAGGTCGACATCATAGATGACAACTTTATCCTGAGGTG----AGGAGCGATGAGTCTGTCGGGAATGTGACTTTTTCATTCGAATATCAAAAGTATGTGACTCTACTTA | 136 | deletion |
| 434 | TGGAAAAATTCTAAAATCTCCTCAAAAAGTAGAGGTCGACATCATAGATGACAACTTTATCCTGA----GAACAGGAGCGATGAGTCTGTCGGGAATGTGACTTTTTCATTCGATTATCAAAAGTATGTGACTCTACTGA | 136 | deletion |
| 435 | TGGAAAAAATCTAAAATCTCCTCAAAAAGTAGAGGTCGACATCATAGATGACAACTTTATCCTGAGGTG----AGGAGCGATGAGTCTGTCGGGAATGTGACTTTTTCATTCGATTATCCAAAGTATGTGACTCTACTTA | 136 | deletion |
| 436 | TGGAAAAAATCTAAAATCTCCTCAAAAAGTAGAGGTCGACATCATAGATGACAACTTTATCCT----TAGAACAGGAGCGATGAGTCTGTCGGGAATGTGACTTTTTCATTCGATTATCAAAAGTATGTGACTCTACTTA | 136 | deletion |
| 437 | TGGAAAAAATCTAAAATCTCCTCAAAAAGTAGAGGTCGACATCATAGATGACAACTTTATCCTGA----GAACAGGAGCGATCAGTCTGTCGGGAATGTGACTTTTTCATTCGATTATCAAAAGTATGTGACTCTACTTA | 136 | deletion |
| 438 | TGGAAAAAATCTAAAATCTCCTCAAAAAGTAGAGGTCGACATCATAGATGACAACTTTATCCTGAGGTG----AGGAGCGATGGGTCTGTCGGGAATGTGACTTTTTCATTCGATTATCAAAAGTATGTGACTCTACTGA | 136 | deletion |
| 439 | TGGAAAAAATCTAAAATCTCCTCAAAAAGTAGAGGTCGACATCATAGATGACAACTTTATCCTGAGGTG----AGGAGCGATGAGACTGTCGGGAATGTGACTTTTTCATTCGATTATCAAAAGTATGTGACTCTACTTA | 136 | deletion |
| 440 | TGGAAAAAATCTTAAATCTCCTCAAAAAGTAGAGGTCGACATCATAGATGACAACTTTATCCTGA----GAACAGGAGCGATGAGTCTGTCGGGAATGTGACTTTTTCATTCGATTATCAAAAGTATGTGACTCTACTTA | 136 | deletion |
| 441 | TGGAAAAAATCTAAAATCTCCTCAAAAAGTAGAGGTCGACATCATAGATGACAACTTTATCCTGA----GAACAGGAGCGATGAGTTTGTCGGGAATGTGACTTTTTCATTCGATTATCAAAAGTATGTGACTCTACTGA | 136 | deletion |
| 442 | TGGACAAAATCTAAAATCTCCTCAAAAAGTAGAGGTCGACATCATAGATGACAACTTTATCCTGA----GAACAGGAGCGATGAGTCTGTCGGGAATGTGACTTTTTCATTCGATTATCAAAAGTATGTGACTCTACTTA | 136 | deletion |
| 443 | TGGAAAAAATCTAAAATCTCCTCAAAAAGTAGAGGTCGACATCATAGATGACAACTTTATCCTGAGGTG----AGGAGCGATTAGTCTGTCGGGAATGTGACTTTTTCATTCGATTATCAAAAGTATGTGACTCTACTTA | 136 | deletion |
| 444 | TGGAAAAAATCTAAAATCTCTTCAAAAAGTAGAGGTCGACATCATAGATGACAACTTTATCCTGAGGTG----AGGAGCGATGAGTCTGTCGGGAATGTGACTTTTTCATTCGATTATCAAAAGTATGTGACTCTACTGA | 136 | deletion |
| 445 | TGGAAAAAATCTAAAATCTCCTCAAAAAGTAGAGGTCGACATCATAGATGACAACTTTATCCTGA----GAACAGGAGCGATGAGTCTGTCGGGAATGTGACTTTTTCATTCGATTACCAAAAGTATGTGACTCTACTGA | 136 | deletion |
| 446 | TGGAAAAAATTTAAAATCTCCTCAAAAAGTAGAGGTCGACATCATAGATGACAACTTTATCCTGAGGTG----AGGAGCGATGAGTCTGTCGGGAATGTGACTTTTTCATTCGATTATCAAAAGTATGTGACTCTACTGA | 136 | deletion |
| 447 | TGGAAAAAATCTAAAATCTCCTCAAAAAGTAGAGGTCGACATCATAGATGACAACTTTATCCTGAGGTG----AGGAGCGATGAGTCTGTAGGGAATGTGACTTTTTCATTCGATTATCAAAAGTATGTGACTCTACTTA | 136 | deletion |
| 448 | TGGAAAAAATCTAAAATCTCCTCAAAAAGTAGAGGTCGACATCATAGATGACAACTTTATCCTGA----GAACATGAGCGATGAGTCTGTCGGGAATGTGACTTTTTCATTCGATTATCAAAAGTATGTGACTCTACTTA | 136 | deletion |
| 449 | TGGAAAAAATCTAAAATCTCCTCAAAAAGTAGAGGTCGACATCATAGATGACAACTTTATCCTGA----GAAAAGGAGCGATGAGTCTGTCGGGAATGTGACTTTTTCATTCGATTATCAAAAGTATGTGACTCTACTTA | 136 | deletion |
| 450 | TGGAAAAAATCTAAAATCTCCTCAAAAAGTAGAGGTCGACATCATAGATGACAACTTTATCCTGA----GAACAGGAGCGATGAGTCTGTCGGGAATGTGACTTTTTCATTCGATTATCAAAAGAATGTGACTCTACTTA | 136 | deletion |
| 451 | TGGAAAAAATCTAAAATCTCCTCAAAAAGTAGAGGTCGACATCATAGATGACAACTTTATCCTGA----GAACAGGAGTGATGAGTCTGTCGGGAATGTGACTTTTTCATTCGATTATCAAAAGTATGTGACTCTACTGA | 136 | deletion |
| 452 | TGGAAAAAATCTAAAATCTCCTCAAAAAGTAGAGGTCGACATCATAGATGACAACTCTATCCTGAGGTG----AGGAGCGATGAGTCTGTCGGGAATGTGACTTTTTCATTCGATTATCAAAAGTATGTGACTCTACTTA | 136 | deletion |
| 453 | TGGAAAAAATCTAAAATCTCCTCAAAAAGTAGGGGTCGACATCATAGATGACAACTTTATCCTGAGGTG----AGGAGCGATGAGTCTGTCGGGAATGTGACTTTTTCATTCGATTATCAAAAGTATGTGACTCTACTGA | 136 | deletion |
| 454 | TGGAAAAAATCTAAAATCTCCTCAAAAAGTAGAGGTCGACATCATAGATGACAACTTTATCCTGA----GAACAGGAGCGTTGAGTCTGTCGGGAATGTGACTTTTTCATTCGATTATCAAAAGTATGTGACTCTACTTA | 136 | deletion |
| 455 | TGGAAAAAATCTAAAATCTCCTCAAAAAGTAGAGGTCGACATCATAGATGACAACTTTATCCTGAGGTG----AGGAGCGATGAGTCTGTCGGGAATGTGACTTTTTCATTTGATTATCAAAAGTATGTGACTCTACTGA | 136 | deletion |
| 456 | TGGAAAAAATCTAAAATCTCCTCAAAAAGTAGAGGTCGACATCATAGATGACAACTTTATCCTGAGGTG----AGGAGCGATGAGTCTGTCGGGAATGTGACTTTATCATTCGATTATCAAAAGTATGTGACTCTACTTA | 136 | deletion |
| 457 | TGGAAAAAATCTAAAATCTCCTCAAAAAGTAGAGGTCGACATCATAGATGACAACTTTATCCTGA----GAACAGGATCGATGAGTCTGTCGGGAATGTGACTTTTTCATTCGATTATCAAAAGTATGTGACTCTACTTA | 136 | deletion |
| 458 | TGGAAAAAATCTAAAATCTCCTCAAAAAGTAGAGGTCGACATCATAGATGACAACTTTATCCTGA----GAACAGGAGCGATGAGTCTGTCGGGAATGTGACTTTTTCATTCGATTATCAAAAGTATGGGACTCTACTTA | 136 | deletion |
| 459 | TGTAAAAAATCTAAAATCTCCTCAAAAAGTAGAGGTCGACATCATAGATGACAACTTTATCCTGA----GAACAGGAGCGATGAGTCTGTCGGGAATGTGACTTTTTCATTCGATTATCAAAAGTATGTGACTCTACTTA | 136 | deletion |
| 460 | TGGAATAAATCTAAAATCTCCTCAAAAAGTAGAGGTCGACATCATAGATGACAACTTTATCCTGAGGTG----AGGAGCGATGAGTCTGTCGGGAATGTGACTTTTTCATTCGATTATCAAAAGTATGTGACTCTACTTA | 136 | deletion |
| 461 | TGGAAAAAATCTAAAATCTCCTCAAAAAGTAGAGGTCGACATCATAGATGACAACTTTATCCTGAGGTG----AGGAGCGATGAGTCTGTCGGGAATGTGACTTTTTCATTCGATTCTCAAAAGTATGTGACTCTACTTA | 136 | deletion |
| 462 | TGGAAAAAATCTAAAATCTCCTCAAAAAGTAGAGGTCGACATCATAGATGACAACTTTATCCTGAGGTG----AGGAGCGATGAGTCTGTCGGGAATGTGACTTTTTCATTCGATTAGCAAAAGTATGTGACTCTACTTA | 136 | deletion |
| 463 | TGGAAAAAATCTAAAATCTCCTCAAAAAGTAGAGGTCGACATCATAGATGACAACTTTATCCTGA----GAACAGGAGCGATGAATCTGTCGGGAATGTGACTTTTTCATTCGATTATCAAAAGTATGTGACTCTACTGA | 136 | deletion |
| 464 | TGGAAAAAACCTAAAATCTCCTCAAAAAGTAGAGGTCGACATCATAGATGACAACTTTATCCTGA----GAACAGGAGCGATGAGTCTGTCGGGAATGTGACTTTTTCATTCGATTATCAAAAGTATGTGACTCTACTTA | 136 | deletion |
| 465 | TGGAAAAAATCTAAAATCTCCTCAAAAAGTAGAGGTCGACATCATAGATGACAACTTTATCCTGTGGTG----AGGAGCGATGAGTCTGTCGGGAATGTGACTTTTTCATTCGATTATCAAAAGTATGTGACTCTACTTA | 136 | deletion |
| 466 | TGGAAAAAATCTAAAATCTCCTCAAAAAGTAGAGGTCGACATCATAGATGACAACTTTATCCTGA----GAACAGGAGCGATGAGTCTGTCGGGAATGTGACTTTTTCATTCGATTATCAAAATTATGTGACTCTACTTA | 136 | deletion |
| 467 | TGGAAAAAATCTAAAATCTCCTCAAAAAGTAGAGGTCGACATCATAGATGACAACTTTATCCTGA----GAACAGGAGCGATGAGACTGTCGGGAATGTGACTTTTTCATTCGATTATCAAAAGTATGTGACTCTACTTA | 136 | deletion |
| 468 | TGGAAAAAATCTAAAATCTCCTCAAAAAGTAGAGGTCGACATCATAGATGACAACTTTATCCTGA----GAACAGGAGCGATGAGTCTGTCGGGAATGTGACTTTTTCATTCGATT-TCAAAAGTATGTGACTCTACTTA | 135 | deletion |
| 469 | TGGAAAAAATCTAAAATCTCCTCAAAAAGTAGAGGTCGACATCATAGATGACAACTTTATCCTGAGGTG----AGGAGCGATGAGTCTGTCGGGAATGTGACTTTTTCATTCGATTATCAAAAGTATGAGACTCTACTTA | 136 | deletion |
| 470 | TGGAAAAAATCTAAAATCTCCTCAAAAAGTAGAGGTCGACATCATAGATGACAACTTTATCCTGA----GAACAGGTGCGATGAGTCTGTCGGGAATGTGACTTTTTCATTCGATTATCAAAAGTATGTGACTCTACTTA | 136 | deletion |
| 471 | TGGAAAAAATCTAAAATCTCCTCAAAAAGTAGAGGTCGACATCATAGATGACAACTTTATCCTGA----GAACAGGAGCGATGAGTCTGTCGGGAATGTGACTTTTTCATTCGATTAACAAAAGTATGTGACTCTACTTA | 136 | deletion |
| 472 | TGGAAAAAATCTAAAATCTCCTCAAAAAGTAGAGGTCGACATCATAGATGACAACTTTATCCTGAGGTG----AGGAGCGATGAGTCTGTCGGGAATGTGACTTTTTCATTCGATTATCAAAAGTATGTGACTCAACTTA | 136 | deletion |
| 473 | TGGAAAAAATCTAAAATCTCCTCAAAAAGTAGAGGTCGACATCATAGATGACAAC-TTATCCTGAGGTG----AGGAGCGATGAGTCTGTCGGGAATGTGACTTTTTCATTCGATTATCAAAAGTATGTGACTCTACTTA | 135 | deletion |
| 474 | TGGAAATAATCTAAAATCTCCTCAAAAAGTAGAGGTCGACATCATAGATGACAACTTTATCCTGAGGTG----AGGAGCGATGAGTCTGTCGGGAATGTGACTTTTTCATTCGATTATCAAAAGTATGTGACTCTACTTA | 136 | deletion |
